# RNA-seq analyses of gene expression in the microsclerotia of *Verticillium dahliae*

**DOI:** 10.1186/1471-2164-14-607

**Published:** 2013-09-09

**Authors:** Dechassa Duressa, Amy Anchieta, Dongquan Chen, Anna Klimes, Maria D Garcia-Pedrajas, Katherine F Dobinson, Steven J Klosterman

**Affiliations:** 1 United States Department of Agriculture – Agricultural Research Service, Salinas, CA, USA; 2 Comprehensive Cancer Center & Division of Preventive Medicine, University of Alabama at Birmingham, Birmingham, AL, USA; 3 Department of Biology, University of Western Ontario, London, ON, Canada; 4 Agriculture and Agri-Food Canada, London, ON, Canada; 5 Department of Physiological and Biological Science, Western New England University, Springfield, MA, USA; 6 Instituto de Hortofruticultura Subtropical y Mediterránea “La Mayora”-Consejo Superior de Investigaciones Científicas (IHSM-UMA-CSIC), Estación Experimental “La Mayora”, Algarrobo-Costa, Málaga, Spain

**Keywords:** *Verticillium dahliae*, Morphogenesis, Microsclerotia, RNA-seq, Gene expression

## Abstract

**Background:**

The soilborne fungus, *Verticillium dahliae*, causes Verticillium wilt disease in plants. Verticillium wilt is difficult to control since *V. dahliae* is capable of persisting in the soil for 10 to 15 years as melanized microsclerotia, rendering crop rotation strategies for disease control ineffective. Microsclerotia of *V. dahliae* overwinter and germinate to produce infectious hyphae that give rise to primary infections. Consequently, microsclerotia formation, maintenance, and germination are critically important processes in the disease cycle of *V. dahliae*.

**Results:**

To shed additional light on the molecular processes that contribute to microsclerotia biogenesis and melanin synthesis in *V. dahliae*, three replicate RNA-seq libraries were prepared from 10 day-old microsclerotia (MS)-producing cultures of *V. dahliae*, strain VdLs.17 (average = 52.23 million reads), and those not producing microsclerotia (NoMS, average = 50.58 million reads). Analyses of these libraries for differential gene expression revealed over 200 differentially expressed genes, including up-regulation of melanogenesis-associated genes tetrahydroxynaphthalene reductase (344-fold increase) and scytalone dehydratase (231-fold increase), and additional genes located in a 48.8 kilobase melanin biosynthetic gene cluster of strain VdLs.17. Nearly 50% of the genes identified as differentially expressed in the MS library encode hypothetical proteins. Additional comparative analyses of gene expression in *V. dahliae*, under growth conditions that promote or preclude microsclerotial development, were conducted using a microarray approach with RNA derived from *V. dahliae* strain Dvd-T5, and from the amicrosclerotial *vdh1* strain. Differential expression of selected genes observed by RNA-seq or microarray analysis was confirmed using RT-qPCR or Northern hybridizations.

**Conclusion:**

Collectively, the data acquired from these investigations provide additional insight into gene expression and molecular processes that occur during MS biogenesis and maturation in *V. dahliae*. The identified gene products could therefore potentially represent new targets for disease control through prevention of survival structure development*.*

## Background

*Verticillium dahliae* is a soilborne, plant pathogenic fungus that causes wilt disease on over 200 plant species worldwide. Verticillium wilt diseases are also referred to as vascular wilts since the fungus invades the plant xylem, disrupting water transport and causing characteristic leaf wilt symptoms. Control of Verticillium wilt diseases is complicated by the lack of genetic resistance in many plant hosts, and also by the persistence of *V. dahliae* in the soil [1].

*Verticillium dahliae* produces melanized survival structures, microsclerotia, that are able to survive in the soil for at least 10 years [2,3]. Root exudates provide a signal for microsclerotial germination [4,5], and upon germination infectious hyphae emerge from the microsclerotia to initiate plant root infection. Following penetration through the plant root epidermis, and an extended period of colonization in which plants are asymptomatic, microsclerotia are produced in large quantities within the dying and necrotic plant tissues, and are subsequently returned to the soil with plant debris to initiate a new round of the disease cycle [1].

Wild-type microsclerotia of *V. dahliae* are characterized by the presence of dense black melanin deposits that appear as granules within and exterior to the cell wall [6]. This melanin is proposed to act as an anti-desiccant and protect against temperature extremes and enzymatic lysis [6], and is synthesized via a dihydroxynaphthalene (DHN) melanin biosynthesis pathway [6,7]. The initial substrate molecule in the DHN pathway is 1,3,6,8-tetrahydroxynaphthalene (1,3,6,8-THN), derived by cyclization of acetate molecules by polyketide synthase (PKS) activity. DHN monomers are generated from the 1,3,6,8-THN through alternating reduction and dehydration reactions involving several intermediates, including scytalone, 1,3,8-THN (trihydroxynaphthalene), and vermelone [6]. The DHN monomers are subsequently oxidized and polymerized into melanin by laccases [6,8]. Though the microsclerotia are typically darkly pigmented, the processes of melanin production and microsclerotial development in *V. dahliae* can be uncoupled [9].

Because of their central role in pathogen survival and initiating plant root infection, the microsclerotia of *V. dahliae* have been considered important targets for disease control [10,11]. This suggestion is supported by the correlation between reduced microsclerotial and pigment production, and reduced survival of *V. dahliae*[12,13]. The results of the analyses of multiple *Agrobacterium tumefaciens*-mediated transformation (ATMT) insertional mutants of *V. dahliae* also indicate an important link between microsclerotial formation and developmental processes needed for virulence [14,15]. For example, a strain of *V. dahliae* with mutation of the stress-responsive glutamic acid-rich protein encoding-gene (*VdGarp1*; *VDAG_08781*) shows both decreased virulence on cotton, and severely reduced microsclerotia formation under nutrient limiting conditions [14]. In contrast, however, *vdpkaC1* mutants exhibit both reduced virulence, and under certain growth conditions enhanced microsclerotia production [16]. The *vdh1* mutant, which exhibits an amicrosclerotial phenotype under both nutrient-rich and starvation growth conditions, is not compromised in virulence [17]. Taken together, these data indicate that microsclerotial development is not always coupled with virulence.

Analyses of gene expression in *Verticillium dahliae* during microsclerotia formation have been carried out previously by comparisons of expressed sequence tag (EST) libraries generated from *V. dahliae* cultures in which microsclerotia were developing (DMS), and those generated from cultures grown in simulated xylem fluid medium (SXM), which does not stimulate microsclerotia development [18]. Those EST analyses revealed in the DMS library expression of pigment synthesis genes, and increased expression of transporter type proteins encoded by several SXM library genes. With the availability of the genomic sequence and the transcriptome of strain VdLs.17 of *V. dahliae* via the Broad Institute [19], more in-depth analysis of gene expression during microsclerotia formation in *V. dahliae* can be conducted using the high-throughput, deep coverage RNA sequencing (RNA-seq) technology. As an additional resource, bioinformatic analyses have enabled categorization of gene sets in *V. dahliae*, strain VdLs.17 [19]. These gene sets include sequences encoding predicted secreted proteins, secondary metabolite synthesis genes, cysteine-rich proteins, and a set of 354 proteins encoded in four ~ 300 kb lineage (VdLs.17)-specific “genome islands”, that showed a significantly increased rate of expression relative to the core genome sequence [19]. All of these individually categorized gene sets can be useful for rapid data-mining in projects employing high throughput analyses of gene expression.

The aim of this study was to identify genes that are differentially expressed in the melanized microsclerotia (MS) of *V. dahliae*. To accomplish this aim, RNA-seq libraries were generated from MS-producing (MS +) cultures of strain VdLs.17, and from cultures of VdLs.17 not producing MS (NoMS), and analyzed for differential gene expression. Additional comparative analyses of gene expression, under growth conditions that promote or preclude microsclerotial development, were conducted using a microarray approach, with RNA derived from *V. dahliae* strain Dvd-T5 and an amicrosclerotial mutant (*vdh1* strain) derived from Dvd-T5 by targeted mutagenesis [20]. Taken together, these results yielded additional insights into gene sets that are of likely importance to the processes involved in melanin production in *V. dahliae*, as well as in MS formation and maintenance, or inhibition of these processes.

## Results

### RNA-seq analyses

**Figure 1:**
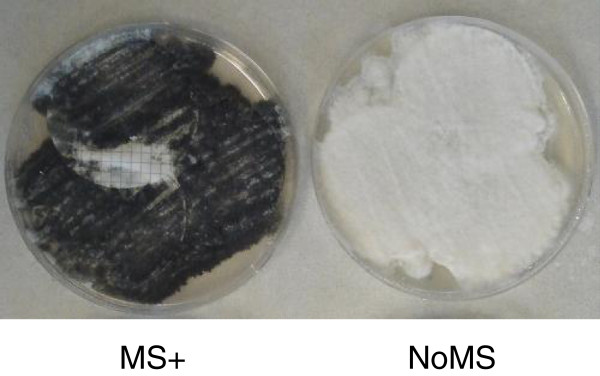
**Ten day-old cultures of *Verticillium dahliae *strain VdLs17, that produced microsclerotia (MS+) or did not produce microsclerotia (NoMS).** Both cultures were grown on potato dextrose agar overlaid with nitrocellulose membranes and incubated at 25°C.

Microsclerotia formation in *Verticillium dahliae* is a default process that occurs as cultures age, and on the potato dextrose agar (PDA) used for the RNA-seq analysis is typically initiated after 3–4 days growth. However, during our studies of *V. dahliae* we have noted that on occasion cultures may spontaneously become amicrosclerotial (K.F. Dobinson, S.J. Klosterman, unpublished). We considered this phenomenon as an opportunity that could be exploited for the analysis of microsclerotial development. Two different RNA-seq approaches were therefore used to assess differential gene expression between MS + and NoMS PDA-grown cultures (Figure 1) of *V. dahliae.* In the first, a genome-wide analysis (GWA) was performed using untrimmed RNA-seq library sequences and FDR-corrected P values (see Methods), and differences were evaluated in gene expression between MS + and NoMS libraries. This method detected a total of 145 differentially expressed genes, 64 of which were up-regulated, and 81 down-regulated in the MS + versus NoMS cultures (Additional files 1 and 2). GWA was also carried out using trimmed RNA sequence reads without applying FDR correction to P values, and selecting genes having > 10-fold up- or down-regulation (listed in Additional file 3). This latter approach resulted in the detection of increased numbers of up-regulated genes involved in melanin synthesis (Additional file 4), a hallmark feature of MS maturation [6]. Specifically, nine pigment synthesis genes related to fungal melanin biosynthesis were identified by data mining analysis (DMA) as up-regulated in the MS + library vs. NoMS library (Additional file 4, #s 1–9), whereas only two of these genes were detected with the first GWA (Additional file 1, #s 1–2). The results of this second GWA (GWA2) were therefore a focal point for this study and were combined into the DMA (Additional file 3) as described below.

The DMA was conducted with 12 sets of genes of interest (GOIs) (Additional file 3). One set of genes examined in this analysis was created by inclusion of the selected genes from GWA2 having > 10-fold up- or down-regulation (Additional file 3). The other 11 gene sets were obtained either from categories of *V. dahliae* genes identified in previous analyses [19], or from multigene families (such as PKSs, NRPSs, hydrophobins, and THN reductases) identified in this study as containing differentially expressed genes (Table 1; Additional file 3). All genes in each set were analyzed for differential expression between the MS + and NoMS libraries, using 10-base end-trimmed RNA sequence reads, and employing common significance criteria of FDR-corrected P values < 0.05, and expression fold change of at least 1.5-fold up- or down-regulation. This analysis yielded a total of 210 differentially expressed genes, 153 of which were up-regulated (Additional file 4) and 57 down-regulated (Additional file 5). Those genes with >20 fold changes in differential expression are shown in Table 2.

**Table 1:** **Differential gene expression counts for data mining analyses of the *Verticillium dahliae *genome**

**Gene category**	**Total number in *V. dahliae *genome**	Differentially expressed*		
		**Upregulated**	**Downregulated**	**Total**
Hydrophobins	5	0	3	3
Tetrahydroxynapthalene reductase	3	3	0	3
Vascular wilt specific genes	14	4	0	4
Cytochrome P450s	69	3	0	3
LS region genes	354	41	6	47
ABC type transporters	35	4	1	5
MFS type transporters	272	13	2	15
Predicted secretome	780	40	33	73
Polyketide synthases	11	2	0	2
Small cysteine rich proteins	258	14	11	25
NRPSs	8	0	0	0

*Differential expression between MS + and NoMS libraries.

**Table 2:** **Genes > 20 fold up- or down-regulated in microsclerotia forming (MS +) vs non microsclerotia forming (NoMS) cultures of *Verticillium dahliae *as revealed by data mining analysis of RNA-seq data**

**Functional category/gene ID**	Expression fold-change* (up-regulated genes)	**Protein name/functional annotation**
**Pigment synthesis**		
VDAG_03665	344.05	Tetrahydroxynaphthalene reductase/melanin biosynthesis
VDAG_03393	231.29	Scytalone dehydratase/melanin biosynthesis
VDAG_04954	165.00	Pigment biosynthesis protein Ayg1
VDAG_00190	136.66	Conidial yellow pigment biosynthesis polyketide synthase/melanin synthesis
VDAG_00189	110.81	Laccase/melanin biosynthesis
VDAG_05181	86.98	Tetrahydroxynaphthalene reductase/melanin biosynthesis
VDAG_00183	40.79	Versicolorin reductase/Polyketide/melanin or aflatoxin biosynthesis
VDAG_00184	23.13	Amino acid adenylation/polyketide synthase
**General metabolism**		
VDAG_03650	246.84	Cytochrome P450 2C3/oxidizes steroids, fatty acids, xenobiotics
VDAG_03079	60.61	Catalase/involved in oxidative stress relief
VDAG_09583	20.18	Alcohol oxidase
**Transporters**		
VDAG_02154	31.65	RTA1/lipid translocating exporter/drug resistance protein
**Cytoskeleton/cell adhesion**		
VDAG_04170	23.44	Keratin associated protein-10
**Hypothetical proteins**		
VDAG_01806	251.76	Unknown
VDAG_00621	201.14	Unknown
VDAG_03732	97.43	Unknown
VDAG_02042	67.51	Unknown
VDAG_03078	60.72	Unknown
VDAG_07349	58.42	Unknown
VDAG_02389	42.48	Unknown
VDAG_08973	37.13	Unknown
VDAG_05569	30.02	Unknown
VDAG_05179	29.71	Unknown
VDAG_10456	28.13	Unknown
VDAG_00592	27.17	Unknown
VDAG_04171	24.46	Unknown
VDAG_06885	22.53	Unknown
VDAG_09869	20.88	Unknown
VDAG_00490	20.38	Unknown
**Cell death**		
VDAG_00261	72.35	IDI-3/induced during incompatibility/cell death
**Functional category/gene ID**	Expression fold-change* (down-regulated genes)	**Protein name/functional annotation**
**General metabolism**		
VDAG_02162	46.09	Oviduct spcefic glycoprotein/glycosyl hydrolase family
VDAG_03507	39.98	Aldo-keto reductase/yakc
VDAG_04322	31.66	Acetylcholinesterase/hydrolyze acetylcholine
VDAG_08741	29.50	Endochitinase
VDAG_06138	24.36	6-hydroxy-D-nicotine oxidase
**Hypothetical proteins**		
VDAG_03287	272.06	Unknown
VDAG_03216	42.95	Unknown
VDAG_03585	38.51	Unknown
VDAG_04032	36.48	Unknown

*FDR P value <0.05 and fold change > 1.5.

In many instances, the recorded magnitude of gene expression fold-change was greater in the DMA than in the GWA (Additional file 6). In DMA, the largest fold-change among the up-regulated and down-regulated genes in the MS + library was recorded for *VDAG_03665* (encoding THN reductase, 344-fold, Table 2), and *VDAG_03287* (encoding hypothetical protein, Table 2), respectively. The DMA also enabled detection of additional pigment synthesis genes expected to be identified in the MS + library, as indicated above.

The number of up- and down-regulated genes observed in DMA of the various categories of *V. dahliae* genes is summarized in Table 1. Among the 11 categories, predicted secretome, LS region protein-encoding genes, and small cysteine-rich protein-encoding genes represented the largest fractions of genes differentially expressed between the two libraries. Functional classification of the differentially expressed genes detected with DMA revealed that over half encode hypothetical proteins of unknown function, with proteins involved in cell metabolism and transport functions representing the second and third largest category, respectively (Additional file 7).

### Functional categories of differentially expressed genes

The lists of genes identified as differentially expressed during microsclerotia development were functionally annotated *in-silico* to identify genes encoding the different types of proteins that are potentially involved in microsclerotia biogenesis. Among these were genes coding for pigment biosynthesis and secondary metabolic enzymes, as well as those coding for cell growth, morphogenesis, signaling and transcription factors associated with pigment production and/or nutrient acquisition, and carbohydrate active enzymes and transport proteins. Below we describe in more detail some of the genes identified as differentially expressed in each one of these major categories.

### Pigment synthesis and secondary metabolism

**Figure 2:**
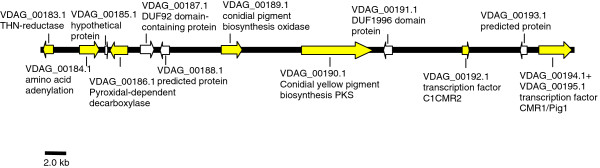
**A 48.8 kb melanin biosynthetic cluster of genes in *Verticillium dahliae*.** Genes up-regulated in the MS + culture are highlighted in yellow.

A number of known melanogenesis-related enzyme-encoding genes were identified by the data mining analysis as up-regulated in MS + culture vs. the NoMS culture, including THN reductase (*VDAG_03665*, 344-fold; *VDAG_05181* 87-fold), scytalone dehydratase (*VDAG_03393*, 231-fold), pigment biosynthesis protein Ayg1 (*VDAG_04954*, 165-fold), conidial yellow pigment biosynthesis PKS (*VDAG_00190*, 137-fold), two laccases (*VDAG_00189*, 111-fold, and *VDAG_00034*, 7-fold), versicolorin reductase (*VDAG_00183*, 41-fold), polyketide synthase (*VDAG_00184*, 23-fold), (Table 2, Additional file 4, #1-9). Among these, those encoding the PKS (*VDAG_00190*), versicolorin reductase (*VDAG_00183*), laccase (*VDAG_00189*) and a second polyketide synthase (*VDAG_00184*) are found clustered in a region spanning 48.8 kb on chromosome 2, supercontig 1 of the *V. dahliae* strain VdLs.17 genome (http://www.broadinstitute.org/annotation/genome/verticillium_dahliae) (Figure 2).

Microsclerotia formation in fungi involves dramatic morphological changes and activation of biosynthetic pathways involved in pigment synthesis [21], processes that are expected to entail transcriptional activation of genes of various cellular functions. Consistent with this premise, several transcriptional regulators were observed as up-regulated in the DMA, including some that are known to control melanin biosynthesis (Table 2, Additional file 4, #47-49). Homologs of the Pig1 and CMR1 transcription factors, which have been previously reported to be involved in fungal melanin synthesis [22,23], were both up-regulated 12-fold in *V. dahliae* VdLs.17 (Additional file 4, #47 and #48). These genes share sequence homology, and are located next to each other in the aforementioned 48.8 kb gene cluster (Figure 2). In addition, a novel transcription factor, encoded by *VDAG_00192* and located in the same cluster, was transcriptionally activated in the MS + culture. The *Pig1* and *CMR1* homologs were originally annotated in the *Verticillium* genome sequence (Broad Institute) as two separate genes (*VDAG_00194* and *VDAG_00195*), but are probably encoded by a single gene (Figure 2). This possibility is supported by the finding in the current study of nearly identical RNA-seq expression levels of *VDAG_00194* and *VDAG_00195* (Additional file 3).

Secondary metabolite biosynthesis in fungi is tightly linked with functions of non-ribosomal peptide synthetases (NRPSs), PKSs, and activities of the cytochrome p450 enzymes (CYPs). Of the eight NRPSs identified in the *V. dahliae* genome (see Methods), none were significantly differentially expressed in the MS + vs. NoMS RNA-seq libraries, whereas of the 10 PKS-encoding genes identified in this species, only the two above-mentioned, *VDAG_00184* and *VDAG_00190*, which share homology with fungal genes involved in the secondary metabolic pathway leading to pigment production [24], and are part of the melanin biosynthesis cluster (Figure 2), were detected as differentially expressed. Also, 3/69 of the CYPs were up-regulated in the MS + library (Table 1). Expression of the CYP gene *VDAG_0365*0 was up-regulated 247-fold in the MS + library (Table 2, Additional file 4, #15), and that of *VDAG_08399* was up-regulated 5-fold (Additional file 4, #40). Though its function in *V. dahliae* is currently unknown, BLAST searches revealed that VDAG_08399 shares homology with *O*-methylsterigmatocystin oxidoreductase proteins involved in the conversion of *O*-methylsterigmatocystin to aflatoxins in *Aspergillus* spp. [25].

### Cell growth, morphogenesis and cell death-related genes

A homolog of *IDI-3*, a cell death-associated gene of *Podospora anserina*[26,27], was strongly up-regulated (72-fold) in the MS + culture (Table 2, Additional file 4, #153), as were cell cycle/cytoskeleton-related genes. The keratin-associated protein-10 gene homolog (*VDAG_04170*; up-regulated 23-fold, Table 2, #70) encodes a large (2924 amino acids) cysteine-rich protein, predicted to be extracellular [19] and displaying weak homology with the developmentally regulated conidiospore surface protein Cmp1, from *Trichoderma* sp. [28]. Genes encoding homologs of actin and other cytoskeletal elements that play critical roles in fungal morphogenesis [29] were also differentially expressed in the MS + or NoMS cultures. A gene encoding a homolog of the cortical actin regulatory protein, Asp1, important for polarized cell growth [30], was up-regulated 4-fold in the MS + culture (Additional file 4, #71). There was also down-regulation of other cytoskeletal-related protein genes (Additional file 5, #s 41–43), and of a homolog of *SUN4* (Additional file 5, #27), which in *S. cerevisiae* regulates cell wall morphogenesis and septation [31].

Catalases, whose products mediate the conversion of hydrogen peroxide to O^-^ and H_2_O, have been implicated in oxidative stress protection [32], and in mediating sclerotial differentiation in filamentous fungi [33]. The expression of only one of three genes encoding predicted secreted catalases, *VDAG_03079*, was up-regulated 61-fold in the MS + culture (Table 2, Additional file 4, #16), while the others (*VDAG_04826* and *VDAG_06575*) were neither significantly up- nor down-regulated (Additional file 3).

We observed a 2-fold increase in the expression level of *VdpkaC1* (Additional file 1, # 31; *VDAG_06474*), which encodes the catalytic subunit of a cAMP-dependent kinase that is involved in regulation of microsclerotial development [16].

### Carbohydrate-active enzymes

The genome of *V. dahliae* encodes an abundance of carbohydrate-active enzymes (CAZys) with diverse predicted activities [19], including roles in fungal cell wall remodeling and plant cell wall degradation. CAZy-encoding sequences significantly up-regulated in the MS library included protein-encoding genes endochitinase *VDAG_04833* (Additional file 4, #42), pectate lyase *VDAG_05402* (Additional file 4, #36), alpha-amylase A *VDAG_05976* (Additional file 4, #37), glucan 1,3-beta-glucosidase *VDAG_09744* (Additional file 4, #29), glucanase B *VDAG_10470* (Additional file 4, #31), and chitin deacetylase *VDAG_05660* (Additional file 4, #25). CAZy genes significantly down-regulated included those encoding an alpha-galactosidase A *VDAG_02431* (Additional file 5, #19), a glucan 1,3-beta-glucosidase *VDAG_07185* (Additional file 5, #21), endoglucanase *VDAG_06254* (Additional file 5, #17), glucan endo-1,3-alpha-glucosidase (*VDAG_04101*, 14-fold), and an endochitinase *VDAG_08741* (Additional file 5, #9). The periplasmic trehalase *VDAG_03038* and the glucose-repressive *Grg1*[34] gene homolog *VDAG_01467* were also up-regulated 10- and 13-fold (Additional file 4, #34 and #73, respectively).

### Transport proteins

The data-mining analyses revealed differential expression in the MS + vs. NoMS libraries of genes encoding ATP-binding cassette (ABC) and major facilitator (MFS) transport proteins, and other transporters. In total, 4/35 ABC type proteins were differentially regulated in the MS library, while 15/272 MFS proteins were differentially regulated (Table 1). Of the differentially expressed ABC transporter-encoding genes, the observed magnitudes of the differences in expression were relatively small, from 2.5-fold up- to 2-fold down-regulation. Only one of the ABC type and two of the predicted MFS genes were identified as down-regulated (Table 1). The sugar transport MFS protein-encoding gene *VDAG_08124* (Additional file 4, #53), was strongly induced, with a 17-fold change in expression. Of the additional transporter type genes identified, the RTA1/lipid translocating exporter gene *VDAG_02154* (Table 2, Additional file 4, #51), was up-regulated 31-fold. The sphingolipid long-chain base-response protein (PIL1) gene (*VDAG_04085*), involved in endocytic transport regulation [35], was also up-regulated 12-fold (Additional file 4, #54).

### Hypothetical protein-encoding genes

Approximately 50% of the differentially expressed genes in the MS + library encode hypothetical proteins for which cellular functions are not yet assigned (Additional file 7). Some of these genes were strongly induced in the MS + library with expression fold changes > 50 (Table 2). *VDAG_01806*, up-regulated 252-fold in the MS + library, was also identified as an EST (VD0100E03) in the library derived from a developing microsclerotia (DMS) culture of *V. dahliae* ([17,18]. At the other end of the spectrum, *VDAG_03287* represents one of the strongly down-regulated (272-fold) hypothetical protein-encoding genes in the MS library (Additional file 5, #29).

Protein motif searches (see Methods) were done for each of the hypothetical proteins in a quest for additional functional information. Most of the hypothetical protein encoding genes appeared to have no identifiable domain in protein motif searches, while others possess domains that function in diverse cellular activities, such as a fasciclin (FAS1) extracellular cell adhesion domain (Additional file 4, #84), a cAMP-regulated phosphoprotein/endosulfine-conserved region (Additional file 4, #111) which might be involved in cell signaling, and fungal Zn(2)-Cys(6) binuclear cluster (Additional file 4, #121 and #124) or bZIP domains (Additional file 4, #127) with potential roles in transcription regulation.

### Reverse-transcription quantitative PCR (RT-qPCR) analyses of gene expression in MS and NoMS cultures

**Figure 3:**
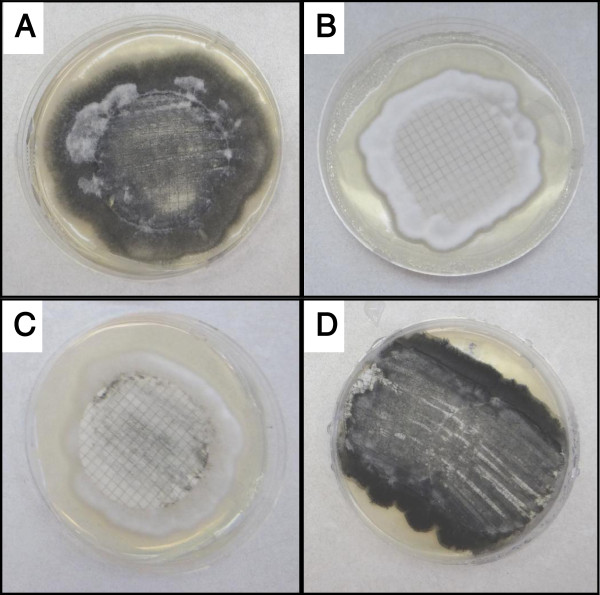
**Examples of cultures of *Verticillium dahliae *that were used to determine relative gene expression by real-time quantitative PCR. A)** A darkly pigmented 12 day culture of *V. dahliae* in which microsclerotia have formed (MS +). **B)** A 12 day culture of *V. dahliae* in which MS have not formed (NoMS). **C)** A 7 day culture of *V. dahliae* in which MS formation has occurred, but at an intermediate level relative to cultures A and D (note the slight pigmentation in C). Cultures A-C were maintained at 25°C. **D)** A culture stored for approximately 6 months at 4°C after an initial incubation period of 4 days at 25°C. The gridded circular nitrocellulose membranes, which allowed easy harvest of the fungal MS and mycelia, are visible in A – C, but obscured in D.

**Figure 4:**
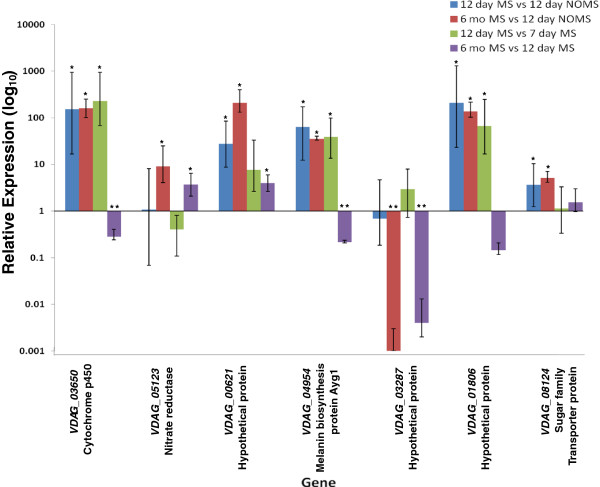
**Taqman-based reverse transcription-quantitative PCR (RT-qPCR) assays of relative gene expression in *Verticillium dahliae*.** The seven genes selected for RT-qPCR experiments were identified by RNA-seq analyses as up- or down-regulated in cultures producing microsclerotia (MS +) or not producing microsclerotia (NoMS) at the sampling time points indicated. Mean expression and error was determined using the Relative Expression Software Tool [58] with ubiquitin and β-tubulin as reference genes (see Methods). Relative expression data from three biological replicates were examined. Error bars indicate 95% confidence intervals. *Up-regulated genes (P < 0.05). **Down-regulated genes (P < 0.05).

Real-time PCR with Taqman assays were conducted to confirm differential expression of seven selected genes in MS + and NoMS cultures of *V. dahliae* (different culture types selected for this purpose are shown in Figure 3). These seven genes included known and hypothetical protein coding genes, as indicated in Figure 4. Among these, RNA-seq analyses had revealed that expression of the hypothetical protein encoding gene *VDAG_03287* was strongly down-regulated in the MS + culture as compared to the NoMS culture. The six other genes in this set were up-regulated as determined by the RNA-seq analyses (Additional file 4).

For the selected genes RT-qPCR analyses of relative expression was compared as follows: expression in the darkly pigmented 12 day MS + cultures (12 d Ms +) vs. that in the 12 day pure white NoMS (12 d NoMS) culture, and that in seven day-old cultures that showed an intermediate level of pigmentation (7 d MS +). Expression of the selected genes in darkly pigmented 6 month-old cultures (6 mo MS +) was also quantified and compared with expression in the above mentioned conditions (Figure 3). Culture types subjected to this study where pigment was either present or absent (Figure 3), were analyzed microscopically at each of the selected time points. Microscopic observations confirmed absence of structures resembling microsclerotia in those cultures lacking dark pigmentation (data not shown).

The results of the RT-qPCR analyses in 12 d MS + vs. 12 d NoMS cultures revealed significant up-regulation of *VDAG_03650*, *VDAG_00621*, *VDAG_04954*, *VDAG_01806*, and *VDAG_08124* in the 12 d MS + culture, but showed no significant up- or down-regulation of *VDAG_03287* or *VDAG_05123* (P < 0.05) (Figure 4). However, all of the genes tested that were significantly up-regulated in the MS culture, as determined by RNA-seq analyses, were also shown by RT-qPCR to be significantly upregulated in the 6 month MS + culture compared to that in the 12 d NoMS culture. Comparison of relative expression between the 6mo MS + culture and the 12 d NoMS culture types also revealed significant down-regulation of *VDAG_03287* (P < 0.05) in the former (Figure 4). With exceptions of *VDAG_03287* or *VDAG_05123* in the 12 d MS + vs. 12 d NoMS comparison, fold-changes in gene expression were similar as assessed by both RNA-seq and RT-qPCR (Additional file 8).

As may be expected in the comparison of the expression levels in two 12 d and 7 d MS + cultures, differences in RT-qPCR-assessed relative gene expression were reduced for *VDAG_00621*, *VDAG_04954*, *VDAG_01806*, and *VDAG_08124* (Figure 4). As mentioned, the 7 d MS + cultures exhibited an intermediate level of melanization (Figure 3). Gene expression was markedly reduced for several of the genes when comparing the darkly pigmented 6 month MS + and 12 d MS + cultures. In particular, *VDAG_03650*, *VDAG_03287*, and the known pigment biosynthesis gene *VDAG_04954* were significantly down-regulated in the 6 month MS + culture, relative to the expression seen for the 12 d MS + culture (Figure 4).

### Microarray analyses

In addition to the RNA-seq analyses done for MS + and NoMS cultures of *V. dahliae* strain VdLs.17, a small-scale microarray analysis was conducted in a second laboratory. *V. dahliae* strains and culture conditions used in the microarray and RNA-seq experiments is shown in Additional file 9. This microarray analysis used a second wild type strain, Dvd-T5, and an amiscrosclerotial (*vdh1*) strain, with cultures grown on either a complete medium agar (CMA), or a basal medium agar (BMA) that induces enhanced production of microsclerotia (Additional file 9).

In this analysis genes were identified that were induced during growth on BMA, and/or repressed in *vdh1* cultures, or Dvd-T5 cultures grown in a liquid CM, i.e. in the absence of microsclerotial development. Genes that exhibited such an expression profile belong to the aforementioned categories of genes found by RNA-seq analysis to be induced during microsclerotial development (Additional file 4) – namely, pigment biosynthesis and secondary metabolism genes, transcriptional regulators, and hypothetical genes. Normalized hybridization ratios of specific genes significantly induced during growth on BMA and/or repressed in the *vdh1* strain and during growth in liquid are shown in Table 3. Many of these genes are involved in secondary metabolism, including the following five pigment-biosynthesis protein-encoding genes that were also shown by the RNA-seq analyses to be up-regulated during microsclerotial development: scytalone dehydratase (*VDAG_03393*), versicolorin reductase (*VDAG_00183*), THN reductase (*VDAG_03665*), laccase (*VDAG_00189*), and *Ayg1* (*VDAG_04954*) (Table 3). Also among the secondary metabolism genes that were up-regulated during growth in microsclerotia-promoting conditions in both the arrays and the RNA-seq analysis was the cytochrome p450 gene *VDAG_03650* (Table 3).

**Table 3:** **Microarray analyses of *Verticillium dahliae *genes induced in a wild type strain and repressed in a hydrophobin mutant strain (*vdh1*)**

**EST ID**	**EST match/comments**	**Putative gene ID (BLASTX match)**	**RNA-seq ID**	**Expression ratios**		
				**Control**^**a **^**vs.**		
				**vdh1**^**b**^	**LIQ**^**c**^	**BM**^**d**^
***Genes induced in BM***						
VD0107B12	BQ109916 VD0100E09	SCD1	VDAG_03393	0.22*	0.22*	9.42**
VD0107A01	BQ110396 VD0107A01	Versicolorin reductase	VDAG_00183	0.26*	0.30*	9.59**
VD0104D02	BQ110359 VD0107D07	H4N reductase	VDAG_03665	0.15**	0.19*	7.12**
VD0103C12	BQ110009 VD0101D02	Laccase	VDAG_00189	0.26*	0.30*	8.49**
VD0100C03	BQ109845 VD0100C03	Ayg1	VDAG_04954	0.93	0.84	3.30*
VD0106G02	BQ110282 VD0106G02;	ACE1	–	0.05**	0.45**	2.95**
	VDAG_03150					
VD0104G06		Hypothetical	VDAG_07138	0.32**	0.42**	4.75**
VD0103G07	BQ109914 VD0100E03	Hypothetical	VDAG_01806	0.21**	0.23*	8.47**
VD0108H01		Cytochrome p450	VDAG_03650	0.42**	0.60*	5.50**
VD0102H04	BQ110648 VD0202A11	Hypothetical	VDAG_03287	0.03**	0.63*	2.2*
	BQ109844 VD0100C02					
VD0102H08	BQ110034 VD0102H08;	Acetoacetyl-CoA synthase	–	0.11**	0.61*	2.61*
	VDAG_09589					
***Genes repressed in vdh1, LIQ***						
VD0103C06	BQ110121 VD0103C06	Fatty acid binding protein	–	0.39**	0.63**	1.38
VD0106H02	BQ110674 VD0206C06;	Heat shock protein	–	0.61**	0.58**	2.234
	VDAG_04645					
VD0110G09	BQ110535 VD0110G09	Long chain base protein	VDAG_07480	0.51**	0.70*	1.61
VD0103C11	BQ110150 VD0103C11;	Oxidoreductase 2-nitropropane dioxygenase	VDAG_00590	0.56**	0.65**	1.60
VD0107C10	BQ110341 VD0107C10;	Cellulose growth specific protein	VDAG_04795	0.33**	0.79*	1.80
VD0102C03	BQ109999 VD0102C03	Phenazine biosynthesis protein	VDAG_00005	0.32**	0.71**	1.97
VD0105B02	BQ110136 VD0105B02	Vacuolar sorting associated	VDAG_03362	0.53**	0.71**	1.36

^a^Control: wild-type strain Dvd-T5 grown on complete medium (CM)^,^agar; ^b^*vdh1*: *vdh1* strain (generated from wild type strain Dvd-T5 by targeted mutagenesis) grown on (CM)^,^agar; ^c^wild-type strain (Dvd-T5) grown in liquid CM (LIQ); ^d^wild-type strain grown on basal medium (BM) agar; ** P ≤ 0.01; *P ≤ 0.05.

Among hypothetical genes, two that were up-regulated in the RNA-seq analysis, *VDAG_0713* and *VDAG_01806*, were also represented among the differentially expressed genes identified by the array analysis, with both shown to be induced during growth on BMA. Notably, the microarray analysis showed up-regulation of hypothetical gene *VDAG_03287*, which was, conversely, down-regulated in the RNA-seq analysis (Table 2).

The microarray analysis also identified several differentially expressed genes in *V. dahliae* Dvd-T5 that were not identified in the RNA-seq analysis (Table 3.). Among these was the transcriptional regulator gene *ACE1* (VD0110G09; *VDAG_03150*), which in Dvd-T5 was repressed in cultures that were not producing MS, and induced during MS development. A cellulose growth specific protein encoding gene (*VDAG_04795*), was also repressed under non- microsclerotial producing conditions, as were two genes with possible roles in lipid metabolism, an acetyl CoA synthetase gene (*VDAG_09589*), and a fatty acid binding protein-encoding gene (VD0103C06; *VDAG_00137*).

### Northern blot validation of microarray and RNA-seq data

The expression of four genes, identified by RNA-seq as up-regulated in the MS + cultures of strain VdLs.17, and by microarray analyses in microsclerotial-producing cultures of strain Dvd-T5, was validated by Northern blot analyses (Additional file 10). These genes, which included two hypothetical protein encoding genes (*VDAG_07138 and VDAG_01806*) and two pigment biosynthesis genes (SCD1; *VDAG_03393* and versicolorin reductase: *VDAG_00183*) were highly expressed at four days post-inoculation in wild-type cultures of *V. dahliae* grown on BMA, but suppressed in a *vdh1* mutant strain of *V. dahliae* grown on CMA (Table 3), and during growth of strain Dvd-T5 in liquid CM. Northern blot analysis also verified that the hypothetical gene *VDAG_03287*, which was down-regulated in the RNA-seq analysis, was up-regulated in four-day old BMA-grown cultures (Additional file 10). The expression profiles of two genes, identified as differentially expressed in the microarray analysis but not in the VdLs.17 RNA-seq analysis, verified by Northern analysis; both the ACE1 transcriptional regulator gene *VDAG_03150* and the fatty acid binding protein *VDAG_00137* were induced in Dvd-T5, and repressed in both the *vdh1* mutant and in liquid-grown cultures (Additional file 10).

Ten genes were shown by microarray analysis to be strongly induced in strain Dvd-T5 during microsclerotia development. To evaluate inter-experimental results the expression of the genes as determined by microarray analysis was compared to that as assessed by Northern blot analysis, RNA-seq and RT-qPCR (Additional file 11). Of the ten genes, eight were also up-regulated in the MS + PDA cultures of strain VdLs.17 as compared to those in the RNA-seq analysis that were not producing MS on PDA (Additional file 11). The exceptions were *VDAG_03287*, which was shown as down-regulated by both RNA-seq and RT-qPCR, but up-regulated by both microarray and Northern blot analyses, and *VDAG_03150* which was, as mentioned above, not detected as differentially expressed by RNA-seq (Additional file 11) in strain VdLs.17 under the conditions tested (Additional file 9).

## Discussion

Microsclerotia are critically important in the disease cycle of *V. dahliae*, and their development has been the subject of intense research for decades [6,12,13,17,18,36-38]. Though significant advances have been made in understanding the morphological and biochemical aspects of microsclerotia biogenesis, the molecular mechanisms underpinning the formation of these structures are still not well understood [39]. The aim of the current study was to identify genes that are differentially expressed in microsclerotia-producing (MS +) and NoMS cultures of *V. dahliae*, to shed further light on these molecular mechanisms. To conduct this work, we took advantage of genome-wide gene expression technologies (RNA-seq and microarray analyses), and also of the recently sequenced genome of *V. dahliae* strain VdLs.17 [19] for *in silico* functional annotation of the genes we identified as differentially expressed during MS development.

The RNA-seq analyses revealed a number of differentially expressed genes including those that encoded enzymes previously known to be involved in the fungal DHN-melanin biosynthetic pathway, including polyketide synthases, THN reductases, scytalone dehydratases, and laccases, [6,7,40,41]. Since MS maturation culminates with the synthesis and deposition of melanin granules in cell walls and intercellular spaces [37], the identification of the highly expressed genes encoding melanin biosynthetic-like enzymes indicated that the RNA-seq libraries were appropriate for evaluation of gene expression during MS maturation.

In the microarray analysis pigment biosynthesis-encoding genes likewise formed the largest group of genes up-regulated during microsclerotial production, and showing high levels of expression, an expression pattern that was confirmed by Northern blot analyses of two of the putative melanin biosynthesis gene orthologs, *VDAG_03393* and *VDAG_00183*. Moreover, pigment biosynthesis gene expression was similarly evident in an earlier EST study of *V. dahliae* cultures undergoing microsclerotia development [18]. Collectively, the overall consistency of these results signals the soundness of the experimental designs used herein.

Although the general biochemical scheme/pathway of fungal DHN melanin biosynthesis is well defined based on experimental evidence gleaned from genetic and biochemical studies [42], the exact identities, functions and multiplicity of all genes/enzymes involved in melanin biosynthesis, and the possible variation of function among species or strains has yet to be determined. The melanin synthesis pathway is complex, involving many regulatory, secretory and structural genes, a complexity that was made apparent by the identification of nine melanin synthesis-related genes that were detected in the RNA-seq study as highly expressed in the MS + culture. Some of these synthetic genes and putative regulators of melanin biosynthesis are clustered together in a 48.8 kb gene region of the *V. dahliae* genome. Similar melanin biosynthetic gene clusters are present in other fungi [22,24,43], and such clusters often include one or more transcription factors, as found in this study.

The RT-qPCR analyses provided confirmation of differential gene expression between MS + and NoMS cultures, and further enabled analyses of gene expression at several culture time points. For example the known pigment biosynthesis gene *VDAG_04954*, encoding a protein highly similar to the melanin biosynthesis protein Ayg1 [44], the cytochrome p450 gene *VDAG_03650*, and the hypothetical protein-encoding gene *VDAG_03287*, were significantly down-regulated in the 6 month MS + culture, relative to the expression seen for the 12 day MS + culture. The down-regulation of *VDAG_04954* in the six month 4°C culture may be indicative of completion of pigment synthesis. Similarly, the function of the cytochrome p450 (VDAG_03650) may not be required at this late stage.

Specific genes, such as *VDAG_03650* and *VDAG_04954*, were identified by RT-qPCR analyses as being expressed in the 12 d MS + cultures at higher levels compared to that in the 7 d MS + cultures. That this enhanced expression is suggestive of their roles in MS (survival structure) biogenesis is confirmed by the corresponding genes having previously been identified as ESTs (clones VD0108H01 and VD0100C03, respectively) expressed in DMS cultures [18], and also observed by microarray analysis to be highly expressed during microsclerotia development [20].

Morphological changes that occur during microsclerotia formation are well documented [10,37,38,45]. In the initial stage of MS formation, the hyphae aggregate, swell and form numerous septa. The septate cells enlarge and become spherical, forming clusters of cells by lateral budding, and during this process of MS maturation some cells undergo autolysis, resulting in a mixture, in individual MS, of dead and live cells [37]. The transition from vegetative (hyphal) growth to survival structure formation is expected to involve external and internal signals (morphogenetic triggers), as well as developmental reprogramming of molecular pathways that direct cell division, growth, and death. Intriguingly, the *V. dahliae* gene VDAG_00261, up-regulated 72-fold in the MS + culture, is highly similar to the *Podospora anserina* cell death-related gene *IDI-3*, the expression of which is induced during the vegetative incompatibility reaction [26]. Likewise, *VDAG_01467* was up-regulated (13-fold) in the 10 day MS + culture, and is highly similar to the *P. anserina* gene *Grg1*, which exhibits increased expression in senescent cultures and has been implicated in control of lifespan [34]. Signaling and effector molecules that orchestrate the death of some cells during *V. dahliae* MS biogenesis may thus involve mechanisms similar to those that in other fungi control senescence and cell death during fungal vegetative incompatibility [46,47], with genes such as *VDAG_00261* and *VDAG_01467* involved in the developmental reprogramming of cells during MS maturation. However, the absence in the *Verticillium* group database of homologs of genes encoding other IDI proteins [26], as determined by BLASTP and tBLASTN (data not shown), suggests divergence between *P. anserina* and *V. dahliae* in the molecular mechanisms operating in stress responses and/or cell death control.

The process of sclerotia formation and maturation in filamentous fungi involves active translocation of molecules from hyphae to developing sclerotia, and exudation/excretion, from sclerotia to the cell environment, of various molecules such as water, amino acids, proteins, soluble carbohydrates, fatty acids, and enzymes [21]. Some of these molecules may be required for synthesis of storage compounds that act as energy reserves for sclerotia germination [21], and the observed increased expression in this study of certain transport protein genes, including those of ABC, MFS, and lipid transport systems, might reflect such a scenario.

Secreted carbohydrate-active enzymes (CAZys) may function not only in the disease process, but also in remodeling of fungal cell walls during MS maturation. The beta-1,3-glucanase encoded by *VDAG_10470*, which is up-regulated in the MS + library, may, for example, contribute to cell wall modifications that accompany morphological transitions in MS development, much as beta-1,3-glucanase is involved in morphogenesis in *S. cerevisiae*[48]. In addition, fungal periplasmic trehalases, which convert trehalose to glucose, are up-regulated under nutrient limiting or starvation conditions [49,50], and the observed enhanced expression, in the MS + library, of the putative periplasmic trehalase *VDAG_03038* may therefore reflect depletion of nutrient levels in the actively growing 10-day *V. dahliae* cultures.

It is conceivable too that reduced expression of genes encoding specific cell wall-degrading activities may also be important for MS maturation. The endochitinase gene *VDAG_08741* and the alpha-glucanase sequence *VDAG_02431* (similar to *S. pombe Agn1*) were both down-regulated in the MS library. While the *S. pombe* protein degrades the septum, allowing cell separation [51], such separation may not be necessary in *V. dahliae* microsclerotia, which comprise both chains and clusters of spherical cells [36,45]. The chitin deacetylase encoded by *VDAG_05660*, which is up-regulated in the MS + library, may on the other hand protect the fungus by converting chitin to a de-*N*-acetylated product like that which has previously been shown to be unsuitable as a chitinase substrate [52]. A decrease in chitinolytic enzyme production during MS maturation, with a concomitant increase in cell wall protection, might be expected for production of the long-lived MS.

In addition to the new candidate genes identified by RNA-seq as differentially expressed in the MS library in this study, there was also a pattern of overlap with genes previously identified in the DMS EST library [18], and by the microarray analysis presented here (EMBL-EBI accession A-MEXP-2325). As described above, pigment biosynthesis genes formed one such clearly identifiable group, as did certain hypothetical/unknown genes. Notably, however, one of the hypothetical genes identified in both analyses (*VDAG_03287*) showed expression patterns that seem to be culture age dependent. This gene was shown by both microarray and Northern blot analyses to be up-regulated in 4-day old microsclerotia-producing cultures, but by RNA-seq and RT-qPCR analysis was down-regulated in 10-day and 6 month microsclerotial cultures of VdLs.17. Likewise, the acetyl CoA synthase gene (*VDAG_09589*) was induced in the 4-day old cultures (as shown by microarray analysis), but not in the 10-day old cultures used to produce the RNA-seq libraries. Also, fluxes in the expression levels of certain other genes, like *VDH1*[17], could have occurred at a developmental stage different than that sampled in the RNA-seq study. Such variation in results obtained through RNA-seq vs. microarray and previous EST analyses may also reflect the difference in strains used for the studies (the lettuce isolate VdLs.17 and tomato isolate Dvd-T5, respectively), and/or the different culture conditions under which the fungi were grown prior to harvest (PDA overlaid with nitrocellulose membranes for RNA-seq analysis vs. cellulose membranes overlaid onto BM for microarray and EST analyses). While enhanced expression of cellulolytic enzymes was not expected to be observed in RNA-seq libraries prepared from cultures grown on nitrocellulose membranes, it is possible that starvation of the fungus in the presence of the cellulose may induce expression of genes encoding cell wall degrading enzymes, such as was observed for *VDAG_04795* in the EST analysis [18], and in the present microarray analysis.

## Conclusion

Information on the molecular control of microsclerotia development in *V. dahliae* is currently still sparse [39]. The developmental reprogramming from the normal growth state to survival structure (MS) formation is expected to involve external and internal signals (morphogenetic triggers) as well as unique cell signaling pathway(s), all of which are yet to be identified and elucidated. We envision that future functional characterization of the melanin synthesis and regulatory genes identified in this study, in conjunction with biochemical analyses, will help refine our understanding of the molecular basis of melanogenesis and MS morphogenesis in *V. dahliae*. Some of the genes identified as up- or down-regulated in the MS + vs. NoMS libraries may be involved in the morphogenetic processes that regulate different aspects of microsclerotial development. In addition, similar to the results obtained in previous EST analyses [18], approximately 50% of the genes that we identified by RNA-seq lacked significant similarity to any known genes. Functional analyses of some of these differentially expressed, unknown genes will undoubtedly advance our understanding of *Verticillium* development. The microsclerotia of *V. dahliae* have been considered important targets for disease control [10,11], and gene products identified in this study could potentially represent new targets for disease control through prevention of survival structure development*.*

## Methods

### Fungal cultures for RNA-seq and RT-qPCR analyses

Strain VdLs.17 of *V. dahliae* was initially grown on potato dextrose agar (PDA). A subculture was initiated by spreading conidia and hyphae onto 47 mm nitrocellulose membranes (0.45 μm pore size, Whatman, Maidstone, England), which were overlaid onto PDA plates. The cultures were maintained at 25°C in the dark. After 10 d cultures that formed dark microsclerotia (MS +), and those that did not form microsclerotia (NoMS) (Figure 1), were harvested for RNA-seq analyses (three replicates of each). The 10 d time point was selected based on the existing knowledge that in laboratory cultures of *V. dahliae* MS typically form over a span of 4 to 12 days. In some instances, however, such as in the 10 d NoMS cultures used for RNA-seq in this study, microsclerotia do not form. Additionally, cultures were harvested at different time points for analyses by RT-qPCR, including from a seven day-old culture with intermediate MS production, based on colony color (7 d MS +; such cultures become more darkly pigmented as the MS maturation process nears completion), a 12 d MS + culture, a 12 d NoMS culture, and 6-month-old MS cultures (6 mo MS +) that had been maintained at 4°C for six months before harvesting (Figure 3). For each sample surface conidia were washed from the membranes using 2 ml of deionized water and a cell spreader. For all RNA extractions the nitrocellulose membranes (Whatman) containing hyphae and microsclerotia, harvested at the appropriate time point, were ground to a fine powder in liquid nitrogen using a mortar and pestle.

### RNA-seq library preparation and construction

Total RNA was extracted from the pulverized fungal tissue using a Qiagen RNeasy Kit (Valencia, CA) following the manufacturer’s instructions, including an on-column DNase I digestion. The subsequent quality control steps and library construction were performed by Centrillion Biosciences (Palo Alto, CA). Briefly, total RNA quality was assessed using an Agilent 2100 bioanalyzer and an Agilent RNA 6000 Nano Kit (Agilent Technologies, Santa Clara, CA). All samples had RNA integrity numbers in the range of 6.8 to 7.6. The Ribo-Zero Magnetic Kit (Epicentre, Madison, Wisconsin) was used for an rRNA depletion step, and the samples were concentrated with an RNeasy MinElute Cleanup Kit (Qiagen). The RNA-Seq libraries were prepared with the ScriptSeq v2 RNA-seq Library Preparation Kit (Epicentre). Library validation was accomplished using Agilent DNA 1000 (Agilent Technologies) and Illumina Library Quantification (Kapa Biosystems, Woburn, MA) Kits. The DNA samples were sequenced using HiSeq 2000 (Illumina) at Centrillion Biosciences. The RNA-seq library statistics derived from the MS + and NoMS cultures are shown in Additional file 12.

### Bioinformatics and functional analyses

Raw sequence reads in fastq format were aligned to the reference genome of the VdLs.17 strain of *Verticillium dahliae* (http://www.broadinstitute.org/annotation/genome/verticillium_dahliae) following a workflow of Galaxy installed at the University of Alabama at Birmingham (http://galaxy.uabgrid.uab.edu). Pre-alignment was conducted to determine if end- trimming sequences reads was necessary, based upon read quality scores. The BAM files were generated following RNA-seq workflow of tophat [53], cufflinks and cuffcompare [54]. These BAM files were loaded into Partek Genomics Suite 6.6 (Saint Louis, MO) for normalization and statistical analyses. Briefly, the reads per kb of exon model per million mapped (RPKM)-normalized reads [55] were calculated, and the expression levels of genes estimated [55-57]. Differential expression was determined by ANOVA, as described in the Partek Genomics Suite 6.6 instruction manual. Differentially expressed gene lists were then generated by applying a false discovery rate (FDR)-corrected cut off P value (P) < 0.05.

For an initial genome-wide analysis (GWA), analyses of differential expression were conducted using untrimmed RNA-seq libraries with alignment to the *V. dahliae* transcriptome, and FDR P value correction. Those genes with P < 0.05 and > 1.3 fold differences between libraries were considered significantly up- or down-regulated. A second GWA data set was prepared by RNA-seq analyses of the full *V. dahliae* transcriptome without FDR-correction. For this analysis, the last 10 bp at the tail ends of the reads were trimmed based on a quality score of 30. Those genes that were > 10 fold up- or down-regulated (82 and 21 genes, respectively) in the second GWA data set were reanalyzed along with various subsets of *V. dahliae* genes (Additional file 3), using a data-mining approach (DMA).

For the data-mining analysis (DMA), RNA-seq analyses were performed with trimmed sequence libraries and gene of interest (GOI) subsets, including those identified by the second GWA and having expression levels estimated as described above. With the exception of the PKSs, NRPSs, and hydrophobins, which were identified for this study using BLASTp analyses of the Verticillium Group Database, the GOI sets were characterized previously [19]. All of these sets are listed in GOI (Additional file 3) under the following categories: cytochrome p450s (P450s), LS region genes, Secretome, Major Facilitator Superfamily (MFS), ATP-Binding Cassette (ABC), Cysteine-Rich Proteins, and Vascular wilt-specific proteins. FDR correction was applied to the DMA, with FDR corrected P values < 0.05 considered significant.

To attain additional insight on the functions of those differentially expressed genes encoding hypothetical proteins, Pfam domain searches were performed with protein sequence queries using a motif search (http://www.genome.jp/tools/motif/) and Pfam domain cutoff E-value 10e-6.

### RT-qPCR

*Verticillium dahliae* strain VdLs.17 was cultured at 25°C in the dark, for seven to twelve days, on nitrocellulose membranes (Whatman, Maidstone, England) overlaid onto PDA plates. To harvest, conidia were washed from membranes using 2 ml deionized water and a cell spreader. Membranes were then ground in liquid nitrogen, and total RNA extracted from 100 mg of the ground powder as described above for the RNA-seq library construction. After extraction, samples were further treated with TURBO DNase (Ambion, Austin, TX) at 37°C for 30 minutes. RNA quality was checked using a Nanodrop (Thermo Scientific, Wilmington, DE), and quantified using a Qubit Fluorometer (Invitrogen, Carlsbad, CA).

To identify an appropriate reference gene for *V. dahliae* MS + and NoMS cultures, the RNA-seq data were examined for potential reference genes for relative gene expression. Based on these analyses we determined that the expression of a ubiquitin gene (*VDAG_05595*) was not significantly up- or down-regulated in the MS + culture relative to that of the NoMS culture (Additional file 13). *VDAG_05595* was therefore used as a reference for relative gene expression analyses in accordance with Pfaffl et al. [58]. Although beta-tubulin gene expression was approximately 2-fold down-regulated in the 10 day MS + library, the use of the beta-tubulin gene in combination with *VDAG_05595* as reference genes resulted in no changes in the significance (P < 0.05) of the relative levels of expression for those genes that were classified as up- or down-regulated in the Taqman assays when *VDAG_05595* was used as the only reference gene (data not shown).

The primers and Taqman probe combinations used in this study are listed in Additional file 14. Reverse transcription of 250 ng RNA was performed in 20 μl reactions, with a 5 min. denaturation at 65°C and 0.5 μg oligo-d(T)15 and 0.77 mM dNTPs, followed by chilling on ice for at least five minutes, incubation at 55°C for 45 min with 200 U SuperScript III (Invitrogen), 1X first strand synthesis buffer, 5 mM DTT and 40 U RNAsin (Promega, Madison, WI), and incubation at 70°C for 15 min to inactivate reverse transcription. For the RT-qPCR, 2 μl cDNA was used as template, and reactions were done in 1X Gene Expression Master Mix (ABI; Applied Biosystems, Carlsbad, CA) with 1X Custom Taqman® Gene Expression Assay reagents (900 nM primer, 200 μM Taqman probe) (ABI). Each sample was run in triplicate, as single probe reactions, in a LightCycler 480 II (Roche Molecular Diagnostics, Pleasanton, CA). The reaction profile included an initial 95°C, 10 minute incubation followed by 40 amplification cycles: of 95°C for 15 seconds, and 60°C for 30 seconds. The resulting Cq values were analyzed for relative expression and errors were determined using REST software version 2009 [58]. PCR products were cloned into pCR 4.0-TOPO (Invitrogen) plasmid vector and sequenced to confirm primer specificity. The resulting plasmids, containing the gene sequences subsequently analyzed in Taqman assays, were used in a five-step, ten-fold dilution standard curve (1 ng to 1 pg) to test the primer efficiency. All of the Taqman assays were at ≥100% efficiency (Additional file 14).

### Fungal cultures for microarray analyses

The *V. dahliae* wild-type Dvd-T5 or the *VDH1* mutant strain (*vdh1*) were grown on agar or in liquid media (BM and CM modified from that of Bennett and Lasure [59]). CM contained 1 × nitrate salts (NaNO_3_ (6 g/L), KCl (0.52 g/L), MgSO_4_.7H_2_O (0.52 g/L), KH_2_PO_4_ (1.52 g/L), 1 × trace elements (ZnSO_4_.7H_2_O (0.022 g/L), H_3_BO_3_ (0.011 g/L), MnCl_2_.4H_2_O (0.005 g/L), FeSO_4_.7H_2_O (0.005 g/L), CoCl_2_.6H2O (0.0017 g/L), CuSO_4_.5H_2_O (0.0016 g/L), Na_2_MoO_4_.2H_2_O (.0015 g/L), Na4EDTA (0.05 g/L), glucose (10 g/L), peptone (2 g/L), yeast extract (1 g/L), casamino acids (1 g/L) and 1× vitamin solution (biotin, pyridoxine, thiamine, riboflavin, *p*-aminobenzoic acid, nicotinic acid, all at 0.01% (w/v)). BM contained glucose (10 g/L), sodium nitrate (0.2 g/L), 1× potassium salts (KCl 0.52 g/L, MgSO4 .7H2O 0.52 g/L, and KH_2_PO4 1.52 g/L), 3 μM thiamine HCl, and 0.1 μM biotin. Agar-grown cultures were inoculated by spreading 1×10^5^ spores onto cellulose membranes (Research Products International, Mount Prospect, IL) overlaying the medium. To harvest the cells, the cellophane was lifted from the agar and immediately ground in liquid nitrogen. Liquid-grown cultures were centrifuged (1480 × *g*) for 10 minutes at 4°C to pellet spores and mycelial fragments. The supernatant was removed, and the pelleted fungal material was ground to a fine powder as above, in liquid nitrogen. Total RNA was isolated from the powdered tissue using TRIzol® Reagent (Invitrogen Canada Inc., Burlington, Canada) extraction as previously described [18].

### Microarray preparation and probe labeling

cDNAs used for generating custom microarrays were selected from the *V. dahliae* simulated xylem fluid medium (SXM) and developing microsclerotia (DMS) libraries [18]. Individual cDNAs were amplified from the plasmid DNA by PCR, in 25 μL reactions containing 400 nM universal primers T3 and T7, 50 ng plasmid DNA, 200 μM each dNTP, 2.5 mM MgCl_2_, 200 mM Tris–HCl (pH 8.4), 500 mM KCl, and 0.5 U Platinum Taq polymerase (Invitrogen, Canada Inc., Burlington, Canada). Denaturation of the template at 94°C for 2 minutes was followed by 30 amplification cycles (94°C for 45 sec, 65°C for 45 sec, 72°C for 1 min), with a final 5 min extension period at 72°C. To verify amplification, products were separated by electrophoresis through 0.8% agarose gels, and visualized by staining with 0.5 μg/mL ethidium bromide. Amplification reactions with low product yields were repeated using a 63°C annealing temperature, and amplification reactions resulting in non-specific amplification were repeated at an annealing temperature of 68°C.

Amplification products were purified by filtration through Sephadex G50 columns (Amersham Biosciences, Baie d’Urfé, QC) in 96-well Polyfiltronics Unifilter plates (Fisher Scientific, Toronto, Canada), dried under vacuum at room temperature using a Savant Speedvac Plus SC210 (Global Medical Instrumentation, Ramsey, USA), and re-suspended in 20 μL 50% dimethyl sulfoxide at a final cDNA concentration of approx 200 ng/μL.

Arrays were constructed by printing amplified cDNA targets onto glass FMB cDNA slides (Full Moon BioSystems, Inc., Sunnyvale, CA), using the VP 470 Manual Glass Slide Indexing unit and VP 478 8-pin Glass Slide Replicator (V&P Scientific, Inc. San Diego, CA). Each array consisted of 768 spots in an 18 mm × 36 mm grid, with two adjacent duplicate spots representing each of 373 target genes. Following spotting, slides were air-dried, and DNA fixed to the slide by UV cross-linking.

To prepare labeled probes, total fungal RNA was further purified using the QIAquick RNeasy Kit (Qiagen) according to manufacturer’s protocols. Ten μg purified total RNA was used as template in T7-RNA polymerase-catalyzed linear amplification reactions, using the RiboAmp RNA Amplification Kit (Arcturus, Mountainview, CA) according to supplier’s directions. The resulting anti-sense RNA (aRNA) was quantified by UV spectrophotometry, and used to prepare probes.

Fluorescent dye-labeled probes, were generated by random-primed reverse transcription in the presence of cyanine-3 (Cy-3)-dCTP or cyanine-5 (Cy-5)-dCTP (Amersham Biosciences). Reverse transcription was done using 10 μg RNA and the CyScribe First-Strand cDNA Labelling Kit (Amersham Biosciences), according to the manufacturer’s directions. The CyScribe GFX Purification Kit (Amersham Biosciences) was used to remove unincorporated nucleotides from the Cy-3- or Cy-5-labelled probes, and fluorescent dye frequency of incorporation (FOI; picomoles of incorporated dye × (324.5 ÷ nanograms cDNA probe)) was measured by visible and UV spectrophotometry. Probes with FOIs between 20 and 50 were used in hybridizations.

### Microarray hybridization

Pre-hybridization of slides was done for 20–30 minutes at 55°C in buffer containing 2 × SSC, 0.1% SDS, and 1% BSA. Following pre-hybridization slides were rinsed thoroughly with sterile distilled, deionized water (DDW), and dried using an ArrayIt microarray high-speed centrifuge (TeleChem International, Sunnyvale, CA).

For hybridization, 20 pmoles of each labeled cDNA probe were pooled and desiccated in an Eppendorf Vacufuge 5301 (Eppendorf, Mississauga Canada), re-suspended in 3 μL nuclease-free DDW, and denatured at 90°C for 5 minutes. Thirty two μL FMB cDNA Hybridization buffer (Full Moon BioSystems, Inc.) was added to the probe solution (final volume 35 μL). The probe solution was applied to the slides, which were then covered with glass cover-slips (Fisher Scientific), and placed in a humidified chamber at 42°C, for 18 hours. Slides were then washed for 20 minutes in a 55°C 2× SSC/2% SDS solution, then three × 1 min. in 55°C 0.2× SSC, quickly rinsed in DDW, and dried with a stream of filtered nitrogen gas.

Immediately after hybridization slides were scanned using a laser scanner (Virtek, Waterloo, ON) and processed, at the London Regional Genomics Institute at Robarts Research Institute (London, ON). The resulting raw scanned images of Cy-3 and Cy-5 fluorescence intensities were processed using Arrayvision 6.0 software (Imaging Research, St. Catharines, ON), wherein the local background was subtracted automatically from each spot.

Background-subtracted spot fluorescence values were imported into GeneSpring 6.0 software (Silicon Genetics, Redwood City, CA) for analysis, in which the intensities of replicate spots were averaged and used to calculate the expression ratios between the control and test conditions. Further normalization of the values was done using the Genespring Per Spot functions (data channel divided by control channel). Background filtering was not done, since the high background levels necessitated a high background cut-off that resulted in the elimination of a number of true positives (expression-validated genes; see below) from the analysis.

### Microarray data analysis and validation

Data from duplicate spots and biological and technical replicates were pooled, and average expression values for the 373 genes were filtered to identify genes showing significant changes in gene expression (regulated genes) as determined using Student’s *t*-test or ANOVA (p < 0.01) with log_2_ of normalized values. Among the differentially regulated genes, 42 genes were identified whose expression changed 1.6-fold or more in at least one test condition when compared with the control condition.

Northern hybridization verification of microarray data was carried out using the DIG Filter Hybridization system (Roche Biomolecular Diagnostics), and methods recommended by the manufacturer. Briefly, total RNA (8.5 μg) was electrophoretically separated on denaturing, 1% agarose, 2% formaldehyde gels. RNA was transferred by capillary blotting to positively charged nylon membranes (Roche Biomolecular Diagnostics), and fixed to the membrane by UV cross-linking.

DIG-labeled probes were prepared using as template 1 μg *Eco*RI-linearized cDNA plasmids from the SXM or DMS libraries [18]. Thirty-minute pre-hybridizations and overnight hybridizations were done at 68°C in DIG Easy Hyb (Roche Molecular Diagnostics). High stringency washes were likewise done at 68°C. Probe-target hybrids were visualized by chemiluminescent assay, using the chemiluminescent alkaline phosphatase substrate CSPD according to manufacturer’s directions.

## Availability of supporting data

RNA-seq data and experimental design were deposited in the ArrayExpress database at the European Molecular Biology Laboratory-European Bioinformatics Institute (EMBL-EBI) http://www.ebi.ac.uk/arrayexpress, with accession number E-MTAB-1581. Microarray data and experimental design were also deposited at EMBL-EBI, accession numbers E-MTAB-1793 and A-MEXP-2325, respectively.

## Competing interests

The authors declare that they have no competing interests.

## Authors’ contributions

Wrote the manuscript: SJK, DD; conceived and designed RNA-seq experiments: SJK, DD, DC; conceived and designed RT-qPCR experiments: SJK, AA; conceived and designed microarray and Northern blot experiments: KFD, AK; Identification of genes involved in secondary metabolism: MGP, SJK; data analyses: SJK, DD, MGP, DC, AA, AK, KFD. All authors approved the final version of the manuscript.

## Supplementary Material

Additional file 1 **Genes up-regulated in microsclerotia forming vs non microsclerotia forming culture of *Verticillium dahliae *as revealed by genome-wide analysis (approach 1) of RNA-seq data.** Click here for file

Additional file 2 **Genes down-regulated in microsclerotia forming vs non microsclerotia forming culture of *Verticillium dahliae *as revealed by genome-wide analysis (approach 1) of RNA-seq data.** Click here for file

Additional file 3 Data mining analyses (see file headings for additional details). Click here for file

Additional file 4 **Genes up-regulated in microsclerotia forming vs non microsclerotia forming cultures of *Verticillium dahliae *as revealed by data mining analysis of RNA-seq data.** Click here for file

Additional file 5 **Genes down-regulated in microsclerotia forming vs. non microsclerotia forming cultures of *Verticillium dahliae *as revealed by data mining analysis of RNA-seq data.** Click here for file

Additional file 6 Comparison of fold changes of up-regulated genes from genome-wide analysis (GWA) and data mining analysis (DMA). Click here for file

Additional file 7 Pie chart illustrating genes up- or down-regulated in the RNA-seq MS library. Click here for file

Additional file 8 **Comparison of fold changes observed in RT-qPCR and RNA-seq.** The seven genes were identified by RNA-seq analyses as up- or down-regulated in cultures producing microsclerotia (MS +) or not producing microsclerotia (NoMS). A) Fold change for RT-qPCR was based on a comparison of 12 day MS cultures vs. 12 day NoMS cultures. B) comparison of 6 month MS + cultures vs 12 day NoMS cultures. The fold change values for RNA-seq were derived from the comparison of 10 day MS +cultures vs. 10 day NoMS cultures. Fold change is shown on a logarithmic scale. Click here for file

Additional file 9 ***Verticillium dahliae *strains and experimental parameters of the RNA-seq and microarray analyses.** Click here for file

Additional file 10 **Validation of selected gene expression profiles by Northern hybridization analysis.** RNA was isolated from complete medium (CM) agar-grown *vdh1*, liquid CM-grown WT, basal medium (BM) agar-grown WT, and CM agar-grown WT cultures. Blots were hybridized with DIG-labeled probes prepared from the cDNAs corresponding to the genes indicated. RNA quality and quantity was verified by ethidium bromide staining. Click here for file

Additional file 11 Comparison of one or more of the four methods used to analyze gene expression. Click here for file

Additional file 12 **Statistics for RNA-Seq libraries derived from *V. dahliae *cultures producing microsclerotia (MS +) or not producing microsclerotia (NoMS).** Click here for file

Additional file 13 RNA-seq data for reference gene selection. Click here for file

Additional file 14 Primers and Taqman probes used in RT-qPCR in this study. Click here for file
