# Supplementary material for: RNA-seq analyses of gene expression in the microsclerotia of Verticillium dahliae
Source: BMC Genomics. 2013 Sep 9;14:607. doi: 10.1186/1471-2164-14-607 (PMC3852263; doi:10.1186/1471-2164-14-607)
Supplement: Additional file 1 — Genes up-regulated in microsclerotia forming vs non microsclerotia forming culture of Verticillium dahliae as revealed by genome-wide analysis (approach 1) of RNA-seq data. [file 1471-2164-14-607-S1.doc]

| **Additional File 1**. Genes up-regulated in microsclerotia forming vs non microsclerotia forming culture of *Verticillium dahliae* as revealed by genome-wide analysis (approach 1) of RNA-seq data | | | |
| --- | --- | --- | --- |
| **Functional category/**  **gene ID** | **Expression (fold change)** | | **Protein name /functional annotation** |
| **Pigment synthesis** |  | |  |
| 1. VDAG_00189 | | 74.11 | Laccase/multicopper oxidase |
| 2. VDAG_00190 | | 95.80 | Condia yellow pigment biosynthesis/polyketide synthase |
| **Protein metabolism** | |  |  |
| 3. VDAG_02606 | | 2.07 | Peptidyl-prolyl cis-trans isomerase cyp6/protein folding |
| **Nucleic acid metabolism** | |  |  |
| 4. VDAG_10083 | | 1.90 | Mating- type switching protein swi10/nucleotide excision repair endonuclease |
| 5. VDAG_04354 | | 2.40 | pre-mRNA processing ATP dependent RNA helicase |
| 6. VDAG_02878 | | 2.47 | RNA binding posttranscriptional regulator csx1 |
| 7. VDAG_02940 | | 2.07 | Helicase SWR1/ chromatin unwinding, DNA repair |
| 8. VDAG_07453 | | 3.24 | DNA repair protein rhp41/nucleotide excision repair |
| **General metabolism** | |  |  |
| 9. VDAG_08836 | | 1.56 | PAP2 domain-containing protein/phosphatidic acid phosphatase |
| 10. VDAG_04197 | | 11.19 | Mitochondrial integral membrane protein/ alpha/beta hydrolase family |
| 11. VDAG_07769 | | 4.11 | Phytanoyl-COA dioxygenase family protein/phytanic acid oxidation |
| 12. VDAG_07783 | | 5.31 | alpha-ketoglutarate-dependent taurine dioxgenase/taurine catabolism |
| 13. VDAG_07799 | | 1.68 | GCY protein/ aldo-keto reductase, NADPH dependent oxidoreductase |
| 14. VDAG_03650 | | 173.19 | Cytochrome P450 2C3/oxidizes steroids, fatty acids, xenobiotics |
| 15. VDAG_04584 | | 2.75 | Alpha/beta hydrolase |
| 16. VDAG_03780 | | 5.64 | Clock control-9 protein/glycosyl transferases group1 |
| 17. VDAG_09744 | | 7.15 | Glucan 1,3-beta-glucosidase/pectate lyase |
| 18. VDAG_05783 | | 3.49 | SiPA3/glucosyl transferase-pectin metabolism |
| 19. VDAG_05123 | | 11.38 | Nitrate reductase/molybdopterin cofactor binding oxidoreductase |
| 20. VDAG_03127 | | 2.36 | Lipase/ lipid formation or hydrolysis |
| 21. VDAG_03053 | | 2.45 | Diacylglycerol O-acyltransferase 2B/ triacylglycerol synthesis |
| 22. VDAG_01897 | | 2.20 | Sterol esterase TGL1/ sterol synthesis |
| 23. VDAG_01819 | | 2.69 | Glutamate decarboxylase/ Gamma-aminoacid butyric acid (GABA) synthesis |
| 24. VDAG_08399 | | 3.64 | O-methylsterigmatocystin oxidoreductase/cytochrome P450  oxidoreductase-involved in aflatoxin synthesis |
| 25. VDAG_08026 | | 4.52 | ATP-dependent permease PDR10/ABC-type transporter |
| 26. VDAG_02258 formation and tr ansport | | 5.14 | GTP-binding protein Di-Ras1/ involved in vesicle formation and transport |
| **Transcription activators** | |  |  |
| 27. VDAG_05342 | | 1.79 | Acriflavine sensitivity control protein acr-2 |
| 28. VDAG_06420 | | 1.34 | Peregrin/transcription activation |
|  | |  |  |

| **Additional File 1**. Continued | | |
| --- | --- | --- |
| **Functional category/ gene ID** | **Expression (fold change)** | **Protein name/functional annotation** |
| **Cell signaling** |  |  |
| 29. VDAG_00644 | 2.56 | PDZ domain containing protein/signaling protein domain |
| 30. VDAG_08127 | 1.96 | GYF d GYF domain/proline rich protein-protein interaction motif |
| 31. VDAG_06474 | 2.68 | cAMP-dependent protein kinase |
| 32. VDAG_09958 | 2.49 | 1-phosphatidylinositol-4,5-bisphosphate phosphodiesterase |
| **Cytoskeleton** |  |  |
| 33. VDAG_09839 | 2.09 | Anucleate primary sterigmata protein B/ microtubule associated |
| 34. VDAG_10441 | 2.66 | Cortical actin cytoskeleton |
| **Hypothetical proteins** |  |  |
| 35. VDAG_09869 | 14.66 | Unknown |
| 36. VDAG_01147 | 3.65 | Unknown |
| 37. VDAG_04924 | 5.83 | Unknown |
| 38. VDAG_02390 | 11.07 | Unknown |
| 39. VDAG_04733 | 1.38 | Unknown |
| 40. VDAG_03837 | 3.72 | Unknown |
| 41. VDAG_05283 | 9.77 | Unknown |
| 42. VDAG_05179 | 22.68 | Unknown |
| 43. VDAG_07529 | 3.48 | Unknown/Nulp1-pending protein |
| 44. VDAG_07717 | 2.07 | Unknown |
| 45. VDAG_03258 | 2.02 | Unknown/transmembrane and coiled-coil domain containing  protein |
| 46. VDAG_03077 | 3.87 | Unknown |
| 47. VDAG_01806 | 166.43 | Unknown |
| 48. VDAG_01895 | 6.14 | Unknown |
| 49. VDAG_02002 | 2.38 | Unknown |
| 50. VDAG_01809 | 1.84 | Unknown |
| 51. VDGA_05054 | 2.23 | Unknown |
| 52. VDAG_06332 | 3.57 | Unknown |
| 53. VDAG_00621 | 138.23 | Unknown |
| 54. VDAG_04920 | 2.40 | Unknown/ EthD domain/ essential for ethyl tert-butyl ether degradation |
| 55. VDAG_09868 | 4.17 | Unknown/Sec2P GDP/GTP exchange factor-involved in vesicular transport |
| 56. VDAG_05179 | 22.68 | Unknown/fungal zn(2)-CYs (6) binuclear cluster domain |
| 57 .VDAG_06665 | 2.19 | Unknown/fungal zn (2)-Cys(6) binuclear cluster domain |
| 58. VDAG_05174 | 5.37 | Unknown/fungal zn(2)-Cys (6) binuclear cluster domain |
| 59. VDAG_10270 | 6.03 | Unknown/Acetyltransferase /acylation |
| 60. VDAG_06885 | 15.53 | Unknown/Ankyrin repeat containing protein/protein-protein interaction |

**Additional File 1.** Continued

| **Functional category/ gene ID** | | **Expression (fold change)** | | **Protein name/functional annotation** | |  |
| --- | --- | --- | --- | --- | --- | --- |
| Hypothetical proteins | |  | |  | |  |
| 61 VDAG_09588 | | 1.33 | | Unknown/SH3 (src homology 3)/ signal transduction related to cytoskeleton | |  |
| 62 VDAG_ 09165 | | 8.92 Unknown/cAMP-regulated phosphoprotein /endosulfine conserved domain | | Unknown | |  |
| 63. VDAG_09726 | | 1.74 | | Unknown/autophagy protein Apg17 | |  |
| 64. VDAG_06360 | | 2.65 | | Unknown/C2 domain-containing protein/targets proteins to membrane | |  |
|  |  | |  | |  | |
