# Supplementary material for: RNA-seq analyses of gene expression in the microsclerotia of Verticillium dahliae
Source: BMC Genomics. 2013 Sep 9;14:607. doi: 10.1186/1471-2164-14-607 (PMC3852263; doi:10.1186/1471-2164-14-607)
Supplement: Additional file 2 — Genes down-regulated in microsclerotia forming vs non microsclerotia forming culture of Verticillium dahliae as revealed by genome-wide analysis (approach 1) of RNA-seq data. [file 1471-2164-14-607-S2.doc]

| **Additional File 2.** Genes down-regulated in microsclerotia forming vs non microsclerotia forming culture of *Verticillium dahliae* as revealed by genome-wide analysis (approach 1) of RNA-seq data | | | | | | | | |
| --- | --- | --- | --- | --- | --- | --- | --- | --- |
| **Functional category/**  **gene ID** | **Expression fold change**  **fold change** | | | | | **Protein name/functional annotation** | |  |
| **Protein metabolism** | | |  | | | |  | |
| 1. VDAG_08799 | | | 1.99 | | | | Proteasome component C5 | |
| 2. VDAG_ 04256 | | | 2.14 | | | | Proteome component PUP1 | |
| 3. VDAG_09843 | | | 2.83 | | | | Histone chaperone ASF1 | |
| 4. VDAG_08749 | | | 2.50 | | | | Metacaspase-1/peptidase, regulated cell death | |
| 5. VDAG_05309 | | | 2.31 | | | | Proteome component Y13 | |
| 6. VDAG_07788 | | | 2.15 | | | | Microsomal signal peptidase 18 kDa subunit | |
| 7. VDAG_02924 | | | 2.20 | | | | Proteome component PRE3 | |
| 8. VDAG_03748 | | | 2.11 | | | | Proteasome component PRE6 | |
| 9. VDAG_05786 | | | 2.19 | | | | 60S ribosomal protein L 10a | |
| 10. VDAG_06944 | | | 2.36 | | | | Proteome subunit alpha type-2 | |
| 11. VDAG_02329 | | | 2.11 | | | | 26S proteosome regulatory subunitRPN9 | |
| 12 VDAG_06001 | | | 1.90 | | | | 26S proteasome regulatory subunit rpn 11 | |
| 13. VDAG_10310 | | | 2.13 | | | | 40S ribosomal protein S14 | |
| 14. VDAG_10491 | | | 2.60 | | | | 40S ribosomal protein S26E | |
| 15. VDAG_10352 | | | 2.26 | | | | Proteasome component C1 | |
| **Nucleic acid metabolism** | | |  | | | |  | |
| 16. VDAG_07372 | | | 4.48 | | | | Endonuclease/exconuclease /phosphatase | |
| **General metabolism** | | |  | | | |  | |
| 17. VDAG_ 04453 | | | 1.56 | | | | Phospho-2-dehydro-3-deoxyheptonate aldolase | |
| 18. VDAG_09840 | | | 1.58 | | | | AhpC/TSA family protein/Redoxin | |
| 19. VDAG_04933 | | | 1.65 | | | | NADH –Ubiquinone oxidoreductase 21 kDa | |
| 20. VDAG_03683 | | | 3.04 | | | | GCY protein/ aldo-keto reductase | |
| 21. VDAG_04777 | | | 2.33 | | | | Dolichyl-phosphate mannosyltransferase polypeptide | |
| 22. VDAG_02585 | | | 2.46 | | | | Phosphotyrosine protein phosphatase | |
| 23. VDAG_04045 | | | 2.46 | | | | Ribonucleoside-diphosphate reductase large chain | |
| 24. VDAG_09345 | | | 6.68 | | | | Isoamyl alcohol oxidase | |
| 25. VDAG_03317 | | | 2.04 | | | | NADH:ubiquinone oxidoreductase17.8KD subunit | |
| 26. VDAG_03388 | | | 6.22 | | | | Repressible alkaline phosphatase | |
| 27. VDAG_01881 | | | 1.62 | | | | Vacuolar ATP synthase 16 kDA proteolipid subunit 2 | |
| 28 VDAG_ 02297 | | | 2.67 | | | | Acetyltransferase | |
| 29. VDAG_02201 | | | 2.14 | | | | Biotin synthase | |
| 30 VDAG_ 01703 | | | 3.42 | | | | NADP-dependent L-serine/L-allo-threonine dehydrogenase | |
| 31. VDAG_01957 | | | 3.25 | | | | Ornithine carbamoyltransferase | |
| 32. VDAG_ 06082 | | | 3.19 | | | | Adenine Phosphoribosyltransferase | |
| 33. VDAG_06254 | | | 5.72 | | | | Endoglucanase-5 | |
| 34. VDAG_06240 | | | 7.46 | | | | Phytanoyl-COA dioxygenase | |
| 35. VDAG_06155 | | | 2.10 | | | | Pectate lyase | |
| **Additional File 2** Continued. | | | | | | | | |
| **Functional category/**  **gene ID** | **Expression fold change** | | | | | | **Protein name/functional annotation** | |
| **Transport** | |  | | | | |  | |
| 36. VDAG_00868 | | | | 1.56 | | | Mitochondrial carrier protein YMC1/ membrane transport | |
| 37. VDAG_04095 | | | | 2.58 | | | Coatomer zeta subunit/ protein transport | |
| **Cell signaling** | | | |  | | |  | |
| 38 VDAG_05247 | | | | 2.03 | | | Protein kinase domain –containing protein | |
| 39 VDAG_02337 | | | | 2.72 | | | Hit family protein/protein-protein interaction | |
| 40. VDAG_10221 | | | | 6.04 | | | Kelch domain protein/protein-protein interaction | |
| **Cytoskeleton** | | | |  | | |  | |
| 41. VDAG_00699 | | | | 1.89 | | | Cofilin/GMF family protein, binds to actin monomers | |
| 42. VDAG_10074 | | | | 3.40 | | | Tubulin beta chain | |
| 43. VDAG_03302 | | | | 3.25 | | | Tubulin alpha chain | |
| **Cell division** | | | |  | | |  | |
| 44. VDAG_07917 | | | | 3.14 | | | Kinetochore protein nuf2/ associated with meiotic spindle | |
| **Hypothetical proteins** | | | |  | | |  | |
| 45. VDAG_08756 | | | | 8.92 | | | Unknown | |
| 46. VDAG_00871 | | | | 2.57 | | | Unknown | |
| 47. VDAG_04125 | | | | 5.83 | | | Unknown | |
| 48. VDAG_01365 | | | | 2.17 | | | Unknown | |
| 49. VDAG_05444 | | | | 2.18 | | | Unknown | |
| 50 VDAG_02536 | | | | 5.04 | | | Unknown | |
| 51. VDAG_03646 | | | | 4.37 | | | Unknown | |
| 52. VDAG_04059 | | | | 3.23 | | | Unknown | |
| 53. VDAG_04937 | | | | 4.17 | | | Unknown | |
| 54. VDAG_02746 | | | | 5.52 | | | Unknown | |
| 55. VDAG_09322 | | | | 11.61 | | | Unknown | |
| 56. VDAG_05184 | | | | 9.09 | | | Unknown | |
| 57. VDAG_09992 | | | | 4.68 | | | Unknown | |
| 58. VDAG_09002 | | | | 5.42 | | | Unknown | |
| 59. VDAG_03216 | | | | 68.23 | | | Unknown | |
| 60 VDAG_03337 | | | | 7.55 | | | Unknown | |
| 61. VDAG-09561 | | | | 2.70 | | | Unknown | |
| 62. VDAG-01778 | | | | 2.17 | | | Unknown | |
| 63 VDAG_06103 | | | | 18.86 | | | Unknown | |
| 64. VDAG_06120 | | | | 7.71 | | | Unknown | |
| 65. VDAG_06360 | | | | 2.27 | | | Unknown | |
|  | |  | | | | |  | |
| **Additional File 2** Continued | |  | | | | |  | |
| **Functional category**  **gene ID** | | **Expression fold change** | | | | | **Protein name/functional annotation** | |
| **Hypothetical proteins** | |  | | | | |  | |
| 66. VDAG_06056 | | | | | 9.15 | | Unknown/SUR7 protein/ endocystosis, sporulation/signaling | |
| 67. VDAG_08336 | | | | | 2.58 | | Unknown | |
| 68. VDAG_08377 | | | | | 1.86 | | Unknown | |
| 69. VDAG _04661 | | | | | 2.42 | | Unknown/chaperone domain protein | |
| 70. VDAG_07658 | | | | | 5.38 | | Unknown/ATPase protein assemby (AAA) | |
| 71 VDAG_06788 | | | | | 3.35 | | Unknown/Histone Chaperone domain (CHZ) | |
| 72. VDAG_04055 | | | | | 1.46 | | Unknown/Tc5 transposase DNA binding domain | |
| 73. VDAG_10147 | | | | | 2.03  hybrid | | Unknown/RNase H2 domain / | |
| 74. VDAG_00018 | | | | | 2.85 | | Unknown/Methyltransferase | |
| 75. VDAG_07766 | | | | | 13.84 | | Unknown/transferase | |
| 76. VDAG_01365 | | | | | 2.17 | | Unknown/Sec2p protein/GDP/GTP exchange factor domain | |
| 77. VDAG_08464 | | | | | 5.54  processes | | Unknown/SPO2 protein domain/ involved in mitosis-meiosis | |
| 78. VDAG_07072 | | | | | 4.54 | | Unknown/calcipressin/calcineurin binding protein, calcium signaling | |
| 79. VDAG_02278 | | | | | 11.57 | | Unknown/ankyrin repeats/protein-protein interaction | |
| 80. VDAG_03646 | | | | | 4.37 | | Unknown/HET/heterokaron incompatibility protein | |
| Protein targeting | | | | |  | |  | |
| 81. VDAG_02516 | | | | | 2.03 | | Unknown/Nascent polypeptide-associated complex subunit beta/prevent in appropriate targeting of non-secretory protein to ER | |
