# Supplementary material for: RNA-seq analyses of gene expression in the microsclerotia of Verticillium dahliae
Source: BMC Genomics. 2013 Sep 9;14:607. doi: 10.1186/1471-2164-14-607 (PMC3852263; doi:10.1186/1471-2164-14-607)
Supplement: Additional file 4 — Genes up-regulated in microsclerotia forming vs non microsclerotia forming cultures of Verticillium dahliae as revealed by data mining analysis of RNA-seq data. [file 1471-2164-14-607-S4.doc]

| **Additional File 4.** Genes up-regulated in microsclerotia forming vs non microsclerotia forming cultures of *Verticillium dahliae* as revealed by data mining analysis* of RNA-seq data | | | | |
| --- | --- | --- | --- | --- |
| **Functional category/** | **Expression** | **Protein name/** | |  |
| **gene ID** | **fold change** | **functional annotation** | |  |
| **Pigment synthesis** |  |  | |  |
|  |  |  | |  |
| 1. VDAG_03665 | 344.05 | Tetrahydroxynaphthalene reductase/melanin biosynthesis | |  |
| 2. VDAG_03393 | 231.29 | Scytalone dehydratase/melanin biosynthesis | |  |
| 3. VDAG_04954 | 165.00 | Pigment biosynthesis protein Ayg1 | |  |
| 4. VDAG_00190 | 136.66 | Conidial yellow pigment biosynthesis polyketide synthase/melanin  synthesis | |  |
| 5. VDAG_00189 | 110.81 | Laccase/melanin biosynthesis | |  |
| 6. VDAG_05181 | 86.98 | Tetrahydroxynaphthalene reductase/melanin biosynthesis | |  |
| 7. VDAG_00183 | 40.79 | Versicolorin reductase/Polyketide/melanin or aflatoxin biosynthesis | |  |
| 8 VDAG_ 00184 | 23.13 | Amino acid adenylation/polyketide synthase | |  |
| 9. VDAG_00034 | 7.44 | Laccase-1/ phenol oxidase/melanin biosynthesis | |  |
| **Protein metabolism** |  |  | |  |
|  |  |  | |  |
| 10. VDAG_ 08958 | 13.71 | Chaperone protein hch A | |  |
| 11. VDAG_05967 | 9.98 | Alkaline proteinase/ protein catabolism | |  |
| 12 .VDAG_07407 | 3.33 | Carboxypeptidase Y inhibitor | |  |
| 13. VDAG_06106 | 1.80 | Endoplasmic oxidoreductin-1/ formation and isomerization of  disulifide bond | |  |
| 14 .VDAG_ 02425 | 1.53 | Mitochondrial chaperone BCS1 | |  |
|  |  |  | |  |
| **General metabolism** |  |  | |  |
|  |  |  | |  |
| 15. VDAG_03650 | 246.84 | Cytochrome P450 2C3/oxidizes steroids, fatty acids, xenobiotics | |  |
| 16. VDAG_03079 | 60.61 | Catalase/involved in oxidative stress relief | |  |
| 17. VDAG_09583 | 20.18 | Alcohol oxidase | |  |
| 18. VDAG_05123 | 16.44 | Nitrate reductase | |  |
| 19. VDAG_10513 | 16.30 | Sulfite oxidase | |  |
| 20. VDAG_08103 | 16.14 | Choline dehydrogenase | |  |
| 21 VDAG_07878 | 15.83 | UDP-glucose, sterole transferase | |  |
| 22. VDAG_02829 | 15.12 | Endo alpha-1,4 polygalactosaminidase precursor | |  |
| 23. VDAG_00782 | 14.57 | Versatile peroxidase VPL1/lignolytic oxidase, phenol oxidase | |  |
| 24. VDAG_07180 | 12.60 | NMRAL1 protein/redox sensor | |  |
| 25. VDAG_05660 | 12.19 | Chitin deacetylase/ hydrolyse chitin to chitosan during growth or  autolysis | |  |
| 26. VDAG_02413 | 11.74 | 2R)-Phospho-3-sulfolactate synthase/primary metabolism | |  |
| 27. VDAG_01149 | 11.68 | FAD binding domain containing protein/oxidoreductase | |  |
| 28. VDAG_03820 | 11.20 | Carbonic anhydrase | |  |
| 29. VDAG_09744 | 10.79 | Glucan 1,3-beta-glucosidase/cell wall metabolism | |  |
| 30. VDAG_01323 | 10.59 | NADP dependent malic enzyme/fatty acid biosynthesis | |  |
| 31. VDAG_10470 | 10.50 | Glucanase B/ glucan hydrolysis/cell wall metabolism | |  |
| **Additional File 4.** Continued. | | | | |
| Functional category/ | Expression | | Protein name/ | |
| Gene ID | fold change | | functional annotation | |
|  |  | |  | |
| **General metabolism** |  | |  | |
| 32. VDAG_01591 | 10.48 | | Acetyl hydrolase/pyruvate metabolism | |
| 33. VDAG_07272 | 10.41 | | R etinol dehydrogenase/ Vitamin A metabolism | |
| 34. VDAG_03038 | 10.15 | | Periplasmic trehalase/ hydrolyze trehalose to glucose | |
| 35. VDAG_09970 | 10.09 | | L-serine dehydratase/ catabolise L-serine to Pyruvate | |
| 36. VDAG_05402 | 6.99 | | Pectate lyase/ cell wall metabolism | |
| 37. VDAG_05976 | 6.91 | | Alpha amylase A type-1/2 / carbohydrate metabolism | |
| 38. VDAG_02346 | 6.32 | | Phosphatidylserine decarboxylase proezyme | |
| 39. VDAG_06816 | 5.29 | | Lysophospholipase/phospholipid hydrolysis | |
| 40. VDAG_08399 | 5.24 | | O-methylsterigmatocystin oxidoreductase | |
| 41 .VDAG_04839 | 3.77 | | Nitrilase/ involved in natural product biosynthesis | |
| 42. VDAG_04833 | 3.57 | | Endochitinase/cell wall metabolism | |
| 43. VDAG_03127 | 3.54 | | Lipase/involved in formation or hydrolysis of lipid | |
| 44. VDAG_05094 | 2.33 | | FDA binding domain containing protein/oxidoreductase | |
| 45. VDAG_05357 | 2.05 | | FDA binding domain containing protein/oxidoreductase | |
| 46. VDAG_00367 | 1.94 | | Cytochrome P450 61 | |
| **Transcription activators** |  | |  | |
|  |  | |  | |
| 47. VDAG_00195 | 12.86 | | Pig1/melanin biosyhtesis related transcription factor | |
| 48. VDAG_00194 | 12.78 | | CMR1/melanin biosynthesis related transcription factor | |
| 49. VDAG_00192 | 10.32 | | Transcription factor | |
| 50. VDAG_04843 | 4.38 | | C6 zinc finger protein | |
| **Transporters** |  | |  | |
| 51. VDAG_02154 | 31.65 | | RTA1/lipid translocating exporter/drug resistance protein | |
| 52. VDAG_04828 | 18.25 | | PRY1 protein ( Sterol transport) | |
| 53 VDAG_08124 | 17.54 | | Sugar transporter protein | |
| 54. VDAG_04085 | 12.51 | | Sphingolipid long-chain base-response protein  PIL1/endocytosis | |
| 55. VDAG_01198 | 5.97 | | Drug transport | |
| 56. VDAG_08168 | 5.58 | | Maltose pemease MAL31/carbohydrate transport | |
| 57. VDAG_09167 | 4.88 | | SNF7 family protein/ protein transport to vacuole and lysome | |
| 58. VDAG_07651 | 3.33 | | Glucose/galactose transporter | |
| 59. VDAG_01883 | 3.17 | | Puromycin resistance protein pur8/drug resistance (toxin efflux) | |
| 60. VDAG_09928 | 3.07 | | ATP binding cassette subfamily G member 2/ABC type transporter | |
| 61. VDAG_02182 | 2.87 | | Allantoate permease/ membrane transport | |
| 62. VDAG_02246 | 2.75 | | Polyamine transporter 3 | |

| **Additional File 4.** Continued  Table 1 Continued |  |  |
| --- | --- | --- |
| **Functional category/**  **gene ID** | **Expression fold**  **change** | **Protein name/functional**  **annotation** |
| Transporters |  |  |
| 63. VDAG_06387 | 2.63 | Metal resistance protein YCF1/ Fugal-vacuole type exporting ATPase |
| 64. VDAG_02402 | 2.40 | Cobalt uptake/ cobalt transporter |
| 65. VDAG_07653 | 2.39 | ATP-binding cassette subfamily D member 1/membrane transport |
| 66. VDAG_06725 | 2.37 | Riboflavin transporter MCH 5/ Vitamin B2 transporter |
| 67. VDAG_01371 | 2.14 | ATP binding cassette transport protein |
| 68. VDAG_07043 | 1.73 | Myo- insitol transporter 1 |
| 69. VDAG_00974 | 1.60 | Low- affinity glucose transporter HXT3/sugar transport |
| Cytoskeleton/cell adhesion |  |  |
| 70. VDAG_04170 | 23.44 | Keratin associated protein-10 |
| 71. VDAG_10441 | 3.93 | Cortical actin cytoskeleton protein Asp1 |
| Cell division/cell cycle |  |  |
| 72. VDAG_02664 | 19.08 | Rec8/meiotic recombination protein |
| 73. VDAG_01467 | 13.21 | Glucose repressive protein/ involved in cell division in yeast |
| Hypothetical proteins |  |  |
|  |  |  |
| 74. VDAG_01806 | 251.76 | Unknown |
| 75. VDAG_00621 | 201.14 | Unknown |
| 76. VDAG_03732 | 97.43 | Unknown |
| 77. VDAG_02042 | 67.51 | Unknown |
| 78 .VDAG_03078 | 60.72 | Unknown/NmrA/ transcription repressor/nitrogen metabolite repressor |
| 79. VDAG_07349 | 58.42 | Unknown/Cysteine rich protein |
| 80. VDAG_02389 | 42.48 | Unknown/Ferittin domain protein |
| 81. VDAG_08973 | 37.13 | Unknown/Alpha/beta hydrolase fold domain |
| 82. VDAG_05569 | 30.02 | Unknown/Cupin domain protein |
| 83. VDAG_05179 | 29.71 | Unknown |
| 84 VDAG_10456 | 28.13 | Unknown/Fasciclin (FAS1) extracellular cell adhesion domain |
| 85. VDAG_00592 | 27.17 | Unknown |
| 86. VDAG_04171 | 24.46 | Unknown |
| 87. VDAG_06885 | 22.53 | Unknown |
| 88. VDAG_09869 | 20.88 | Unknown |
| 89. VDAG_00490 | 20.38 | Unknown |
| 90. VDAG_00294 | 18.29 | Unknown |
| 91 VDAG_00587 | 17.59 | Unknown |
| 92. VDGA_06061 | 17.13 | Unknown |

| **Additional File 4.** Continued |  |  |
| --- | --- | --- |
| **Functional category**  **Gene ID** | **Expression fold**  **change** | **Protein name/functional annotation** |
| Hypothetical proteins |  |  |
| 93. VDAG_08109 | 16.64 | Unknown |
| 94 VDAG_02390 | 16.40 | Unknown |
| 95 VDAG_04197 | 16.39 | Unknown/mitochondrial integral membrane protein |
| 96. VDAG_00159 | 16.37 | Unknown |
| 97 .VDAG_10300 | 16.12 | Unknown |
| 98. VDAG_09105 | 15.85 | Unknown |
| 99. VDAG_09172 | 15.51 | Unknown |
| 100. VDAG_10160 | 15.26 | Unknown |
| 101. VDAG_05283 | 15.11 | Unknown |
| 102. VDAG_05172 | 14.84 | Unknown |
| 103. VDAG_08034 | 14.30 | Unknown |
| 104. VDAG_07138 | 14.19 | Unknown |
| 105. VDAG_02705 | 13.38 | Unknown |
|  |  |  |
| 106. VDAG_09165 | 13.23 | Unknown/cAMP regulated phosphoprotein/endosulfine conserved |
| 107. VDAG_10437 | 13.18 | Unknown |
| 108. VDAG_03882 | 12.71 | Unknown |
| 109..VDAG_ 08132 | 12.57 | Unknown/Histidine phosphatase domain protein |
| 110. VDAG_04010 | 11.77 | Unknown |
| 111. VDAG_09145 | 11.43 | Unknown/cAMP regulated phosphoprotein/endosulfine conseved region |
| 112. VDAG_02347 | 11.35 | Unknown |
| 113. VDAG_08640 | 10.67 | Unknown/bZIP transcription factor |
| 114. VDAG_02155 | 10.35 | Unknown |
| 115. VDAG_04395 | 8.65 | Unknown |
| 116. VDAG_04924 | 8.29 | Unknown |
| 117. V DAG_04908 | 8.24 | Unknown |
| 118. VDAG_02713 | 8.05 | Unknown |
| 119. VDAG_02227 | 7.73 | Unknown |
| 120. VDAG_05173 | 7.45 | Unknown |
| 121. VDAG_05174 | 7.17 | Unknown/Fungal zn(2)-Cys (6) binuclear cluster domain |
| 122. VDAG_04912 | 7.17 | Unknown |
| 123. VDAG_09152 | 5.94 | Unknown |
| 124. VDAG_02407 | 5.45 | Unknown/Fungal zn(2)-Cys (6) binuclear cluster domain |
| 125. VDAG_08202 | 5.37 | Unknown |
| 126. VDAG_05793 | 5.19 | Unknown |
|  |  |  |
| **Additional File 4.** Continued  Table 1 Continued |  |  |
| Functional category  Gene ID | Expression fold  change | Protein name/functional annotation |
| Gene ID |  |  |
| Hypothetical proteins |  |  |
| 127. VDAG_02408 | 4.87 | Unknown/ bZIP transcription factor domain protein |
| 128. VDAG_02406 | 3.80 | Unknown |
| 129. VDAG_03711 | 3.45 | Unknown/WSC domain containing protein |
| 130. VDAG_09180 | 3.41 | Unknown |
| 131. VDAG_07195 | 2.78 | Unknown/ could be pathogenesis related protein |
| 132. VDAG_04597 | 2.68 | Unknown/Secretory pathway transport protein domain |
| 133. VDAG_04832 | 2.49 | Unknown/Integerase domain protein |
| 134. VDAG_09166 | 2.41 | Unknown |
| 135. VDAG_02388 | 2.35 | Unknown |
| 136. VDAG_09161 | 2.34 | Unknown /HMG/SAM-box domains containing protein |
| 137. VDAG_06602 | 2.14 | Unknown/ Exportin 1 domain protein ( translocates proteins out of nucleaus) |
| 138. VDAG_01014 | 2.11 | Unknown /major transport facilitator superfamily protein |
| 139. VDAG_03386 | 2.02 | Unknown |
| 140. VDAG_04929 | 1.98 | Unknown |
| 141. VDAG_04872 | 1.97 | Unknown |
| 142. VDAG_04850 | 1.91 | Unknown |
| 143. VDAG_04845 | 1.89 | Unknown |
| 144. VDAG_02428 | 1.85 | Unknown |
| 145. VDAG_02073 | 1. 81 | Unknown |
| 146. VDAG_02256 | 1.80 | Unknown |
| 147. VDAG_05160 | 1.78 | Unknown |
| 148. VDAG_05220 | 1.74 | Unknown |
| 149. VDAG_10077 | 1.65 | Unknown |
| 150. VDAG_07331 | 1.61 | Unknown |
| 151. VDAG_07772 | 1.59 | Unknown |
| 152. VDAG_07148 | 1.57 | Unknown |
| Cell death |  |  |
| 153. VDAG_00261 | 72.35 | IDI-3/ induced during incompatibility/cell death |
|  |  |  |

*FDR p-value <0.005 and fold change >= 1.5
