# Supplementary material for: RNA-seq analyses of gene expression in the microsclerotia of Verticillium dahliae
Source: BMC Genomics. 2013 Sep 9;14:607. doi: 10.1186/1471-2164-14-607 (PMC3852263; doi:10.1186/1471-2164-14-607)
Supplement: Additional file 5 — Genes down-regulated in microsclerotia forming vs. non microsclerotia forming cultures of Verticillium dahliae as revealed by data mining analysis of RNA-seq data. [file 1471-2164-14-607-S5.doc]

| **Additional File 5**. Genes down-regulated in microsclerotia forming vs non microsclerotia forming cultures of *Verticillium dahliae* as revealed by data mining analysis* of RNA-seq data | | | |  |
| --- | --- | --- | --- | --- |
| **Functional category/**  **gene ID** | **Expression (fold change)** | **Protein name/functional annotation** | |  |
| **Pigment synthesis** |  |  | |  |
| 1. VDAG_04798 | 3.86 | Tyrosinase/melanin synthesis | |  |
| **Protein metabolism** |  |  | |  |
| 2. VDAG_03555 | 13.73 | Thymus specific serine protease | |  |
| **Nucleic acid metabolism** |  |  | |  |
| 3. VDAG_05416 | 6.11 | Guanyl-specific ribonuclease F1 | |  |
| 4. VDAG_00107 | 1.88 | Guanyl-specific ribonuclease F1 | |  |
| 5. VDAG_05006 | 2.62 | Endonuclease/Exonuclease /phosphatase- family protein | |  |
| **General metabolism** |  |  | |  |
| 6. VDAG_02162 | 46.09 | Oviduct spcefic glycoprotein/glycosyl hydrolase family | |  |
| 7. VDAG_03507 | 39.98 | Aldo-keto reductase/ yakc | |  |
| 8. VDAG_04322 | 31.66 | Acetylcholinesterase/ hydrolyze acetylcholine | |  |
| 9. VDAG_08741 | 29.50 | Endochitinase | |  |
| 10. VDAG_06138 | 24.36 | 6-hydroxy-D-nicotine oxidase/ degrades nicotine | |  |
| 11. VDAG_00831 | 15.60 | Alkaline phosphatase/dephosphorylation | |  |
| 12. VDAG_07399 | 15.05 | HpcH/Hpal aldolase/citrate lyase family protein | |  |
| 13. VDAG_ 04101 | 13.59 | Glucan endo-1,3 alpha glucosidase agn1/ degrades cell wall around septum | |  |
| 14. VDAG_04551 | 5.03 | Antigen | |  |
| 15. VDAG_05345 | 5.35 | Acid phosphatase PHO1 | |  |
| 16. VDAG_03084 | 4.11 | Alpha/beta hydrolase | |  |
| 17. VDAG_06254 | 3.74 | Endoglucanase-5/cellulose hydrolysis | |  |
| 18. VDAG_04886 | 3.11 | 1-aminocyclopropane-1-carboxylate synthase/ethylene biosynthesis | |  |
| 19. VDAG_02431 | 3.06 | Alpha galactosidase A /glycolipid and glycoprotein hydolysis | |  |
| 20. VDAG_08095 | 2.81 | Phosphoglycerate mutase family protein | |  |
| 21. VDAG_07185 | 2.77 | Glucan 1,3-beta glucosidase | |  |
| 22. VDAG_ 09506 | 2.02 | 5’/3’- nucleotidase SurE family protein /acid phosphatase | |  |
| 23. VDAG_10379 | 1.94 | 3-phytase/phytic acid hydrolysis | |  |
| **Transporters** |  |  | |  |
| 24. VDAG_03336 | 14.35 | Excitatory aminoacid transporter/glutamate transporter | |  |
| 25. VDAG_07186 | 10.92 | Ammonium transporter 1 | |  |
| 26. VDAG_08061 | 3.14  transition | SGE1/major facilitator transport protein family | |  |
| 27. VDAG_07342 | 2.06 | Brefeldin A resistance protein/ ABC type transporter | |  |
|  |  |  | |  |
| **Cell division**  reproduction |  |  | |  |
| 28. VDAG_03021 | 2.31 | Septation protein SUN 4/ cell wall septation | |  |
|  |  |  | |  |
|  |  |  | |  |
|  |  |  | |  |
| **Additional File 5**. Continued | | | |  |
| **Functional category/**  **gene ID** | **Expression (fold change)** | | **Protein name/functional annotation** | |
| **Hypothetical proteins** |  |  | |  |
| 29. VDAG_03287 | 272.06 | Unknown | |  |
| 30. VDAG_03216 | 42.95 | Unknown | |  |
| 31. VDAG_03585 | 38.51 | Unknown | |  |
| 32. VDAG_04032 | 36.48 | Unknown /similar to ATPAP16/PAP 16 | |  |
| 33. VDAG_04102 | 15.88 | Unknown | |  |
| 34 VDAG_05963 | 15.09 | Unknown | |  |
| 35. VDAG_04634 | 13.42 | Unknown | |  |
| 36. VDAG_01833 | 13.23 | Unknown/DUF341 containing protein | |  |
| 37. VDAG_06103 | 12.24 | Unknown | |  |
| 38. VDAG_08653 | 10.77 | Unknown | |  |
| 39. VDAG_07701 | 7.83 | Unknown | |  |
| 40. VDAG_08956 | 7.04 | Unknown/fungal hydrophobin domain containing | |  |
| 41. VDAG_07851 | 6.21 | Unknown/fungal hydrophobin domain containing | |  |
| 42. VDAG_04306 | 6.13 | Unknown/ GPI anchored domain containing protein | |  |
| 43. VDAG_07947 | 5.22 | Unknown | |  |
| 44. VDAG_03544 | 3.98 | Unknown | |  |
| 45. VDAG-06198 | 3.90  domain | Unknown/general substrate facilitator/transporter | |  |
| 46. VDAG-02273 | 3.77 | Unknown/Fungal hydrophobin domain containing  protein | |  |
| 47. VDAG_ 04890 | 3.66 | Unknown | |  |
| 48. VDAG_02746 | 3.52 | Unknown | |  |
| 49. VDAG_04899 | 3.37 | Unknown | |  |
| 50. VDAG_07682 | 3.18 | Unknown | |  |
| 51. VDAG_02343 | 3.16  domain | Unknown/Glycosyl hydrolases family 17 protein | |  |
| 52. VDAG_03886 | 2.82 | Unknown/ allergen Asp f 4 family protein | |  |
| 53. VDAG_03201 | 2.45  pathogenesis | Unknown/ CFEM domain protein/possible role in | |  |
| 54. VDAG_01339 | 2.18 | Unknown | |  |
| 55. VDAG_09209 | 1.97 | Unknown | |  |
| 56. VDAG_09181 | 1.93 | Unknown/NmrA domain protein | |  |
| 57. VDAG_01864 | 1.52 | Unknown/ | |  |
