# Supplementary material for: RNA-seq analyses of gene expression in the microsclerotia of Verticillium dahliae
Source: BMC Genomics. 2013 Sep 9;14:607. doi: 10.1186/1471-2164-14-607 (PMC3852263; doi:10.1186/1471-2164-14-607)
Supplement: Additional file 6 — Comparison of fold changes of up-regulated genes from genome-wide analysis (GWA) and data mining analysis (DMA). [file 1471-2164-14-607-S6.doc]

Additional File 6. Comparison of fold-change differences in expression between the genome-wide analysis (GWA) and data mining analysis (DMA)

| Gene ID*^a^* | Name | GWA*^b^* | DMA |
| --- | --- | --- | --- |
| VDAG_03650 | cytochrome P450 2C3 | 173.20 | 246.84 |
| VDAG_01806 | hypothetical protein | 166.43 | 251.76 |
| VDAG_00621 | hypothetical protein | 138.22 | 201.14 |
| VDAG_00190 | conidial yellow pigment biosynthesis polyketide synthase | 95.80 | 136.66 |
| VDAG_00189 | laccase | 74.11 | 110.81 |
| VDAG_05179 | hypothetical protein | 22.68 | 29.71 |
| VDAG_06885 | hypothetical protein | 15.53 | 22.53 |
| VDAG_09869 | hypothetical protein | 14.66 | 20.89 |
| VDAG_05123 | nitrate reductase | 11.34 | 16.44 |
| VDAG_04197 | mitochondrial integral membrane protein | 11.19 | 16.40 |

*^a^*Gene IDs and names assigned by the Broad Institute

*^b^*Fold change up-regulation in MS vs NoMS libraries
