# Supplementary material for: RNA-seq analyses of gene expression in the microsclerotia of Verticillium dahliae
Source: BMC Genomics. 2013 Sep 9;14:607. doi: 10.1186/1471-2164-14-607 (PMC3852263; doi:10.1186/1471-2164-14-607)
Supplement: Additional file 7 — Pie chart illustrating genes up- or down-regulated in the RNA-seq MS library. [file 1471-2164-14-607-S7.doc]

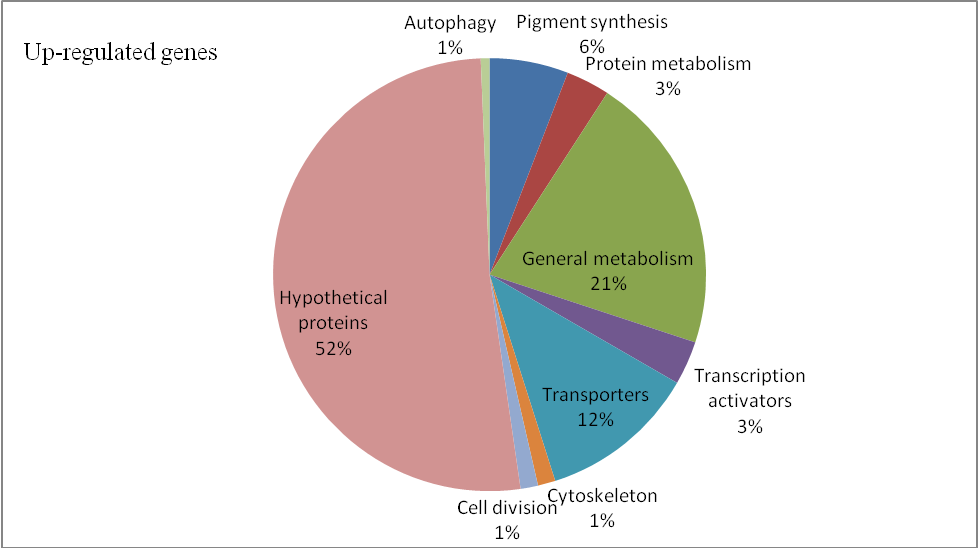


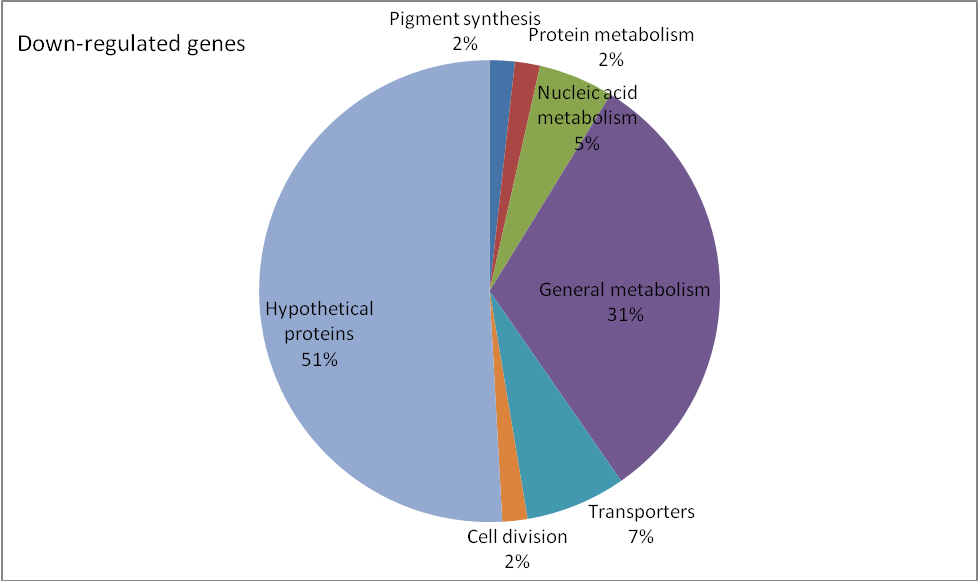


Additional file 7. Functional category of differentially expressed genes detected by data mining analysis.
