# Supplementary figures and images for: RNA-seq analyses of gene expression in the microsclerotia of Verticillium dahliae
Source: BMC Genomics. 2013 Sep 9;14:607. doi: 10.1186/1471-2164-14-607 (PMC3852263; doi:10.1186/1471-2164-14-607)

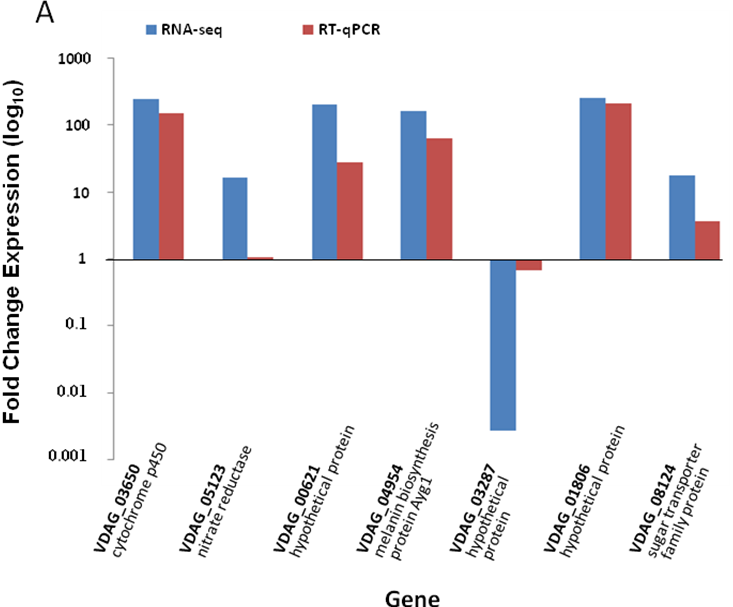


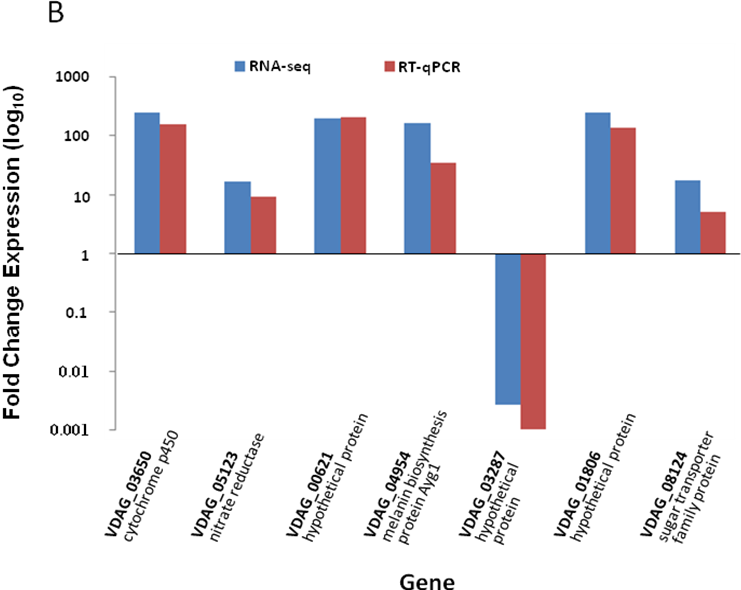

Supplement: Additional file 8 — Comparison of fold changes observed in RT-qPCR and RNA-seq. The seven genes were identified by RNA-seq analyses as up- or down-regulated in cultures producing microsclerotia (MS +) or not producing microsclerotia (NoMS). A) Fold change for RT-qPCR was based on a comparison of 12 day MS cultures vs. 12 day NoMS cultures. B) comparison of 6 month MS + cultures vs 12 day NoMS cultures. The fold change values for RNA-seq were derived from the comparison of 10 day MS +cultures vs. 10 day NoMS cultures. Fold change is shown on a logarithmic scale. [file 1471-2164-14-607-S8.doc]

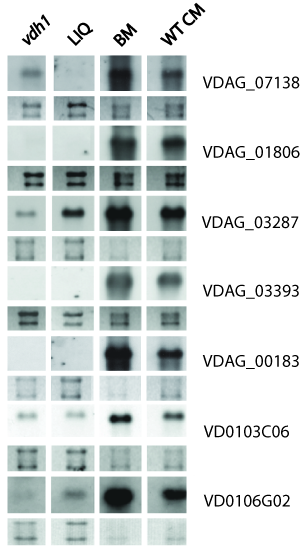

Supplement: Additional file 10 — Validation of selected gene expression profiles by Northern hybridization analysis. RNA was isolated from complete medium (CM) agar-grown vdh1, liquid CM-grown WT, basal medium (BM) agar-grown WT, and CM agar-grown WT cultures. Blots were hybridized with DIG-labeled probes prepared from the cDNAs corresponding to the genes indicated. RNA quality and quantity was verified by ethidium bromide staining. [file 1471-2164-14-607-S10.tiff]
