# Supplementary material for: RNA-seq analyses of gene expression in the microsclerotia of Verticillium dahliae
Source: BMC Genomics. 2013 Sep 9;14:607. doi: 10.1186/1471-2164-14-607 (PMC3852263; doi:10.1186/1471-2164-14-607)
Supplement: Additional file 9 — Verticillium dahliae strains and experimental parameters of the RNA-seq and microarray analyses. [file 1471-2164-14-607-S9.doc]

Additional file 9. Fungal strains and experimental conditions used for transcript accumulation analyses

| **Experiment** | **Fungal strain (genotype)** | **Culture**  **medium** | **Culture phenotype** |
| --- | --- | --- | --- |
| RNA-seq^a^ | VdLs.17 (wt) | PDA | MS production  No MS production |
| RT-qPCR^b^ | VdLs.17 | PDA | MS production  No MS production |
| Microarray^c^ | Dvd-T5 (wt) | CMA | MS production |
|  |  | BMA | Enhanced MS production |
|  | VDAT2-17^d^ (*vdh1*) | CMA | No MS production^a^ |
| Northern blot^c^ | Dvd-T5 | CMA | MS production |
|  |  | BMA | Enhanced MS production |
|  | VDAT2-17 | CMA | No MS production^a^ |

^a^Phenotype analysis at 10 days post-inoculation (dpi) on potato dextrose agar (PDA). ^b^Phenotype analysis at 12 dpi on PDA. ^c^Phenotype analysis at 4 dpi on complete medium agar (CMA) or basal medium agar (BMA). ^d^ VDAT2-17 generated from Dvd-T5 by targeted mutagenesis via transformation with *Agrobacterium tumefaciens* strain carrying transposon-disrupted *VDH1*gene. For additional detail, see Klimes and Dobinson [17].
