# Supplementary material for: RNA-seq analyses of gene expression in the microsclerotia of Verticillium dahliae
Source: BMC Genomics. 2013 Sep 9;14:607. doi: 10.1186/1471-2164-14-607 (PMC3852263; doi:10.1186/1471-2164-14-607)
Supplement: Additional file 11 — Comparison of one or more of the four methods used to analyze gene expression. [file 1471-2164-14-607-S11.doc]

Additional file 11. Comparison of expression for genes highly expressed in basal medium agar (BMA) in microarray analysis vs expression by northern blot, RNA-seq and RT-qPCR

| EST ID/RNA-seq ID | Northern blot^a^ | RNA-seq^b^ | RT-qPCR^c^ |
| --- | --- | --- | --- |
| VD0107B12/ VDAG_03393 | Up* | Up | nt |
| VD0107A01/ VDAG_00183 | Up | Up | nt |
| VD0104D02/ VDAG_03665 | nt | Up | nt |
| VD0103C12/ VDAG_00189 | nt | Up | nt |
| VD0100C03/ VDAG_04954 | nt | Up | Up |
| VD0106G02/ VDAG_03150 | Up | nd | nt |
| VD0104G06/ VDAG_07138 | Up | Up | nt |
| VD0103G07/ VDAG_01806 | Up | Up | Up |
| VD0108H01/ VDAG_03650 | nt | Up | Up |
| VD0102H04/ VDAG_03287 | Up | Down | Down |

^a^Four d wt strain grown on complete medium agar (CMA) vs. four d wt strain grown on basal medium agar (BMA).^b^10 d MS vs. 10 d NoMS wild-type strain (wt) VdLs.17 on potato dextrose agar (PDA); ^c^12 d MS vs. 12 d NoMS wt VdLs.17 on PDA; nd = not detected as differentially expressed; nt = not tested. *Up or down is a reference to genes detected as up- or down-regulated.
