# Supplementary material for: RNA-seq analyses of gene expression in the microsclerotia of Verticillium dahliae
Source: BMC Genomics. 2013 Sep 9;14:607. doi: 10.1186/1471-2164-14-607 (PMC3852263; doi:10.1186/1471-2164-14-607)
Supplement: Additional file 12 — Statistics for RNA-Seq libraries derived from V. dahliae cultures producing microsclerotia (MS +) or not producing microsclerotia (NoMS). [file 1471-2164-14-607-S12.doc]

Additional File 12. Statistics for RNA-Seq libraries derived from *Verticillium dahliae* cultures that produced microsclerotia

(MS) or those that had not produced microsclerotia (NoMS)

| Sample tube # | Description | Average fragment size (bp) | Reads  (Millions) | Total bases (Gb) | Percent reads  mapped* |
| --- | --- | --- | --- | --- | --- |
| 1 | MS culture 1 | 376 | 48.60 | 4.86 | 89.4 |
| 2 | MS culture 2 | 362 | 52.97 | 5.30 | 87.8 |
| 3 | MS culture 3 | 369 | 58.12 | 5.81 | 90.5 |
| 4 | NoMS culture 1 | 368 | 46.82 | 4.68 | 88.3 |
| 5 | NoMS culture 2 | 365 | 46.31 | 4.63 | 90.0 |
| 6 | NoMS culture 3 | 364 | 58.61 | 5.86 | 87.7 |

*Reads mapping to the genome of Verticillium dahliae, strain VdLs.17 at http://www.broadinstitute.org/annotation/- genome/verticillium_dahliae.
