# Supplementary material for: RNA-seq analyses of gene expression in the microsclerotia of Verticillium dahliae
Source: BMC Genomics. 2013 Sep 9;14:607. doi: 10.1186/1471-2164-14-607 (PMC3852263; doi:10.1186/1471-2164-14-607)
Supplement: Additional file 13 — RNA-seq data for reference gene selection. [file 1471-2164-14-607-S13.doc]

Additional File 13. RNAseq data for candidate reference genes

| Gene ID* | Description* | p-value  (MS vs. NoMS) | Fold Change  (MS vs. NoMS) | Fold Change(MS vs. NoMS) |
| --- | --- | --- | --- | --- |
| VDAG_07506 | actin-2 | 0.041047 | 1.19551 | MS up vs NoMS |
| VDAG_08303 | myosin-2 | 0.030053 | 1.44484 | MS up vs NoMS |
| VDAG_00364 | ubiquitin | 0.020779 | -1.66625 | MS down vs NoMS |
| VDAG_05595 | ubiquitin | 0.819263 | -1.46841 | MS down vs NoMS |
| VDAG_10074 | tubulin beta chain | 0.001824 | -2.2962 | MS down vs NoMS |
| VDAG_01827 | tubulin gamma chain | 0.710198 | 1.10368 | MS up vs NoMS |
| VDAG_05517 | elongation factor 1-alpha | 0.031951 | 1.98411 | MS up vs NoMS |
| VDAG_01458 | elongation factor Tu | 0.073911 | -1.44324 | MS down vs NoMS |
| VDAG_08916 | glyceraldehyde-3-phosphate dehydrogenase | 0.095675 | 1.45088 | MS up vs NoMS |

*Gene identity (ID) and descriptions were derived from the Verticillium group website at the Broad Institute (http://www.broadinstitute.org/annotation/genome/verticillium_dahliae).
