# Supplementary material for: RNA-seq analyses of gene expression in the microsclerotia of Verticillium dahliae
Source: BMC Genomics. 2013 Sep 9;14:607. doi: 10.1186/1471-2164-14-607 (PMC3852263; doi:10.1186/1471-2164-14-607)
Supplement: Additional file 14 — Primers and Taqman probes used in RT-qPCR in this study. [file 1471-2164-14-607-S14.doc]

Additional file 14. Primers and Taqman probes used in RT-qPCR in this study

| Gene ID | | Forward (5’ - 3’) | Reverse (5’ - 3’) | Probe (5’ - 3’) | Amplification Efficiency (%) |
| --- | --- | --- | --- | --- | --- |
| VDAG_04954 | CCTGTGGGAGACCAAATGGA | | CGTCATGGAAAGGGTACACACTT | TTGGTGCATGGAAATC | 107 |
| VDAG_05595 | GCTCACCGGTAAGACTATCACA | | TTGGACTTCACATTGTCGATCGT | TTGGAAGTCGAATCATCC | 104 |
| VDAG_00621 | GGGCTCCGGGCAAGTAC | | GTACCACTTGCCGTCATCGTAA | AAGGCGCTGGTACTCC | 100 |
| VDAG_03287 | CTACGTCGCCACCATTGC | | CCACAGACGGAAGCAGTTGAT | CCGCAGCCAGTGCCAG | 109 |
| VDAG_01806 | GGGACGGAGAGATGGATGGAA | | ACCGCAGATTGCCAGTAAGAC | ATGCGCCTCGTCCTGTC | 113 |
| VDAG_03650 | GCGGTGCGTTGCTTCAG | | AGGAAGGTCTGCACAAAGCT | CCGCCGCCCTCCAT | 107 |
| VDAG_08124 | GCCGCGTCATTTGCATCTTC | | CCCAGGATGGTCAGCACAAT | ATGGGCTGCGTCTTC | 100 |
| VDAG_05123 | GGCCGAGTACCGAATCCA | | TGCAGTAGAGCCAGGTTTCG | TTCGACGCGCTGCACC | 112 |
